# Capped Vapor–Liquid–Solid Growth of Vanadium-Substituted Molybdenum Disulfide Ultrathin Films for Enhanced Photocatalytic Activity

**DOI:** 10.1021/acsnano.5c17367

**Published:** 2026-01-07

**Authors:** Pin-Pin Huang, Mohammad Qorbani, Ying-Ti Hung, Ying-Ren Lai, Amr Sabbah, Mao-Feng Tseng, Chih-Yang Huang, Sumangaladevi Koodathil, Septia Kholimatussadiah, Mahmoud Kamal Hussien, Tzu-Hsuan Feng, Yo-Hsun Liu, Hsin Wang, Jia-Wei Lin, Chen-Hao Wang, Chih-I Wu, Michitoshi Hayashi, Kuei-Hsien Chen, Li-Chyong Chen

**Affiliations:** † Institute of Atomic and Molecular Sciences, 38017 Academia Sinica, Taipei 10617, Taiwan; ‡ Department of Chemistry, 34879 National Taiwan Normal University, Taipei 10610, Taiwan; § Undergraduate Program of Electro-Optical Engineering, National Taiwan Normal University, Taipei 11677, Taiwan; ∥ Institute of Electro-Optical Engineering, National Taiwan Normal University, Taipei 11677, Taiwan; ⊥ Center for Condensed Matter Sciences, 33561 National Taiwan University, Taipei 10617, Taiwan; # Center of Atomic Initiative for New Materials, National Taiwan University, Taipei 10617, Taiwan; ∇ Department of Materials Science and Engineering, National Taiwan University, Taipei 10617, Taiwan; ○ Tabbin Institute for Metallurgical Studies, Tabbin, Helwan 109, Cairo 11421, Egypt; ◆ Interdisciplinary Research Center for Hydrogen Technologies and Carbon Management (IRC-HTCM), King Fahd University of Petroleum & Minerals, Dhahran 31261, Saudi Arabia; ¶ School of Electrical, Computer and Energy Engineering, 530268 Arizona State University, Tempe, Arizona 85287-5706, United States; †† Molecular Science and Technology Program, Taiwan International Graduate Program, Academia Sinica, Taipei 11529, Taiwan; ‡‡ International Graduate Program of Molecular Science and Technology, National Taiwan University, Taipei 10617, Taiwan; §§ Department of Physics, National Taiwan University, Taipei 10617, Taiwan; ∥∥ Nano Science and Technology, Taiwan International Graduate Program, Academia Sinica, Taipei 11529, Taiwan; ⊥⊥ Institute of Physics, Academia Sinica, Taipei 11529, Taiwan; ## Department of Chemistry, Faculty of Science, Assiut University, Assiut 71516, Egypt; ∇∇ Graduate Institute of Photonics and Optoelectronics, National Taiwan University, Taipei 10617, Taiwan; ○○ National Center for Theoretical Sciences, Taipei 10617, Taiwan; a Department of Materials Science and Engineering, National Taiwan University of Science and Technology, Taipei 106335, Taiwan

**Keywords:** VLS growth method, 2D materials, semicondcutor, dopant−vacancy pairing, photocatalysis, CO_2_ reduction, scanning electrochemical microscopy

## Abstract

The exceptional and tunable physicochemical properties of 2D transition metal dichalcogenides (TMDCs) have made them model catalysts for fundamental studies and applications. Activating the inert basal plane holds the key to utilizing wafer-scale TMDCs in artificial photosynthesis. To address this challenge, we report a SiO_2_-capped vapor–liquid–solid (VLS) growth method that assists in substituting vanadium into the molybdenum disulfide ultrathin film and introducing sulfur vacancies to form S_vac_-Mo_1–*x*_V_*x*_S_2_. By optimizing the thickness of solid precursors and the SiO_2_-capping layer (membrane layer), as well as the growth temperature, we demonstrate control over the film thickness, vanadium concentration, and film uniformity. Our results reveal the presence of the V–S_vac_ pairs, manifesting in the enhanced S_vac_ concentration and charge density transfer among V–S–Mo atoms, with multifaceted benefits, including increasing light absorption, photoluminescence quenching, crystal structure distortion, efficient binding of CO_2_ or H_2_O on the surface, improved charge transfer/transport, and a suitable energy band diagram. Furthermore, the 2D S_vac_-Mo_1–*x*_V_*x*_S_2_ model catalyst films, with abundant V–S_vac_ pair active sites, exhibit a stable and boosted photocatalytic CO_2_ reduction to CO, specifically yielding ∼5 times more than that of pristine MoS_2_. Our study demonstrates the origin of V–S_vac_ pairs in host MoS_2_, leading to basal plane activation. This suggests a foundation for future research on pairing dopants or alloying elements with defects for efficient photocatalyst design.

## Graphical abstract

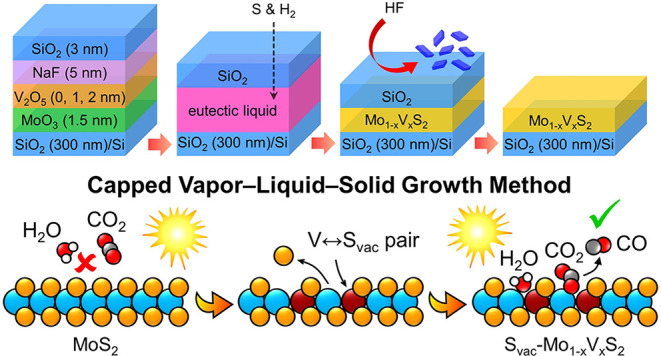

## Introduction

Visible-light-driven photocatalytic (PC) conversion of CO_2_ to value-added C_1_–C_2_ products over semiconductors is a heterogeneous process and one of the ways to meet net-zero emissions.^1–4^ Unfortunately, the overall efficiency of this process over noble-metal-free semiconductors, which is still less than that of the highest-productivity plants with a typical efficiency of ∼1% annually, cannot meet the commercialization requirements.^5–9^ The low PC performance mainly originates from weak light–matter interaction, rapid electron–hole recombination rate, limited photocarrier transport/diffusion length, limited active sites, inappropriate binding energies of reactants and reaction intermediates, unsuitable energy band alignment, and low charge transfer rate to reactants.^10–13^ Therefore, there is still room to understand how each factor plays a role in the PC CO_2_ reduction reaction (CO_2_RR).

2D semiconducting transition metal dichalcogenides (TMDCs) (their general chemical formula is MX_2_, where M and X are transition metal and chalcogen, respectively) have emerged as a new class of nonprecious photocatalysts with tunable optoelectronic properties. In contrast with the catalytic active edge sites,^12,14^ its basal plane is inert, which has motivated scientists to expose the edge by nanostructuring in recent years.^15,16^ Another scenario is to enhance the intrinsic catalytic activity of the basal plane to take advantage of the high surface-to-edge atoms by doping,^17^ alloying,^18^ defect engineering,^19^ introducing adatoms,^20^ applying strain,^21^ and applying an external magnetic field.^22^ Introducing an alloying or doping element is a promising approach because it can regulate the binding energies of reactants as well as the key intermediates, and also modify the optoelectronic properties of the host 2D materials. Nevertheless, there is a need to develop versatile growth methods for reproducible, uniform, and large-area growth of 2D TMDCs and a controllable concentration of dopants and alloying elements. In order to minimize errors originating from the product signals by gas chromatography (GC) spectrometry and area normalization of the product yield, a wafer-scale film is required for the PC CO_2_RR over 2D TMDCs film. Therefore, a bottom-up growth method is necessary.

It is over half a century since the vapor–liquid–solid (VLS) growth method was applied to grow 1D structures, such as nanowires, by precipitation from supersaturated catalytic eutectic liquid droplets.^23–25^ Lately, there has been a growing interest among scientists in using the VLS growth method to synthesize layered van der Waals materials.^26–28^ In 2018, Li et al. reported VLS-grown monolayer MoS_2_ nanoribbons and other TMDCs on the NaCl substrtate.^29^ The growth process, initiated by the reaction between MoO_3_ and NaCl, leads to the formation of liquid-phase Na_2_Mo_2_O_7_ droplets. When saturated with sulfur, these droplets crawl on the surface to minimize the total interfacial free energy, resulting in the growth of ribbons and bilayer regions with different stacking orders and ripple-like features. Later, Chang et al. proposed a self-capping VLS method for growing large-grain and continuous MoS_2_ films on SiO_2_/Si substrate.^30^ The process involved sequential deposition of ultrathin precursor MoO_3_, a diffusion membrane of SiO_2_, and NaF salt layers. At elevated temperatures, MoO_3_ diffused through the SiO_2_ top layer due to the pressure gradient and capillary phenomenon, producing MoO_2_F_2_ gas and forming a eutectic Na_2_Mo_2_O_7_ liquid on the surface via reaction with NaF. Subsequently, sulfurization converted the liquid into MoS_2_ seeds. These MoS_2_ seeds function as a self-capping layer, directing the horizontal growth direction. These observations indicate the potential of the VLS growth method to scale up production of 2D materials. Nonetheless, the wafer-scale utilization of the VLS reaction for doped/alloyed TMDCs remains largely unexplored. If realized, it will be possible to study the atomistic insights of various dopants and alloying elements in TMDC films and apply them to several applications, especially those that need large-scale films.

Among the various dopants and alloying elements in TMDCs, vanadium can adjust its optical, electrical, and magnetic properties, but its impact on catalytic activity is less studied.^31–36^ In current research, significant attention has been directed toward the electrochemical hydrogen evolution reaction (HER). The enhancements in HER performance can be categorized into two primary groups: (i) phase transition (e.g., semiconductor–to–metal 2H–to–1T phase transition) which was mainly observed by the J_1_, J_2_, and J_3_ Raman peaks, the Mo 3*d* orbital splitting between trigonal-prismatic (2H) and octahedral (1T) phases, and scanning transmission electron microscopy (STEM) images of microstructures by other scientists; and (ii) modification of charge transfer capability within the semiconductor phase.^37–42^ Therefore, the potential use of vanadium-incorporated semiconducting TMDCs as photocatalysts is still largely unknown and needs to be systematically investigated.

Here, we present a method to incorporate vanadium in atomically thin hexagonal MoS_2_ and explore the optoelectronic features of the resulting films, focusing on their potential use in gas-phase photocatalytic CO_2_ reduction to address large increases in atmospheric CO_2_ concentrations.^43^ We report a capped (or membrane-controlled) VLS growth method to produce wafer-scale and full-coverage ultrathin vanadium molybdenum disulfide with sulfur vacancy (*i.e.*, S_vac_-Mo_1–*x*_V_*x*_S_2_) alloy films to overcome the limitation of alloying element concentration, a major advancement over our previous work.^30^ The growth parameters, *i.e.*, the thickness of the SiO_2_ capping (or membrane) layer, growth temperature, and the layer thickness of sandwiched solid precursors in nanometer-size space, were optimized systematically. The membrane layer played a triple role, preventing the escape of vanadium-based compounds, supplying sulfur and hydrogen into the eutectic liquid via gas-in-solid diffusion, and assisting symmetric 2D growth of grains. Experimental observations, theoretical calculations, and modeling of atomic configuration reveal a correlation between V–S_vac_ pairs with a significant impact on the optoelectronic properties of grown atomically thin films. As a model photocatalyst, S_vac_-Mo_1–*x*_V_*x*_S_2_ demonstrates an enhanced water-humidified CO_2_ to CO reduction yield of about five times larger than that of the pristine MoS_2_ under artificial sunlight, where vanadium boosts the catalytic activity of S_vac_. This augmented performance is attributed to the presence of catalytic V–S_vac_ pair active sites confirmed by first-principles calculations, modified energy band diagram, enhanced absorption in the visible light region, and enhanced charge transfer or transport, as supported by spatially resolved scanning electrochemical microscopy (SECM).

## Results and Discussion

### Membrane-Controlled VLS Growth

Figure S1 displays the setup of our proposed membrane-controlled VLS growth method and the temperature ramping profiles. We observed that the presence of the membrane layer (or capping layer), growth temperature, and thickness of solid precursors are the main controlling factors, which will be discussed in the following text. In the first step, we successively deposited 1.5 nm MoO_3_, 2 nm V_2_O_5_, and 5 nm NaF layers (solid precursors) by the plasma-enhanced atomic layer deposition (PEALD) and electron beam evaporation (EBE), standing for the noncapped VLS growth (Figures 1a and S2). After the growth process, the X-ray photoelectron spectroscopy (XPS) spectrum does not show a vanadium signal in the V 2*p* window (Figure S3). The significant escape of vanadium can be attributed to the formation of low-boiling-point vanadium fluoride compounds from the reaction between V_2_O_5_ and NaF salt (Figure S4).

**Figure 1:**
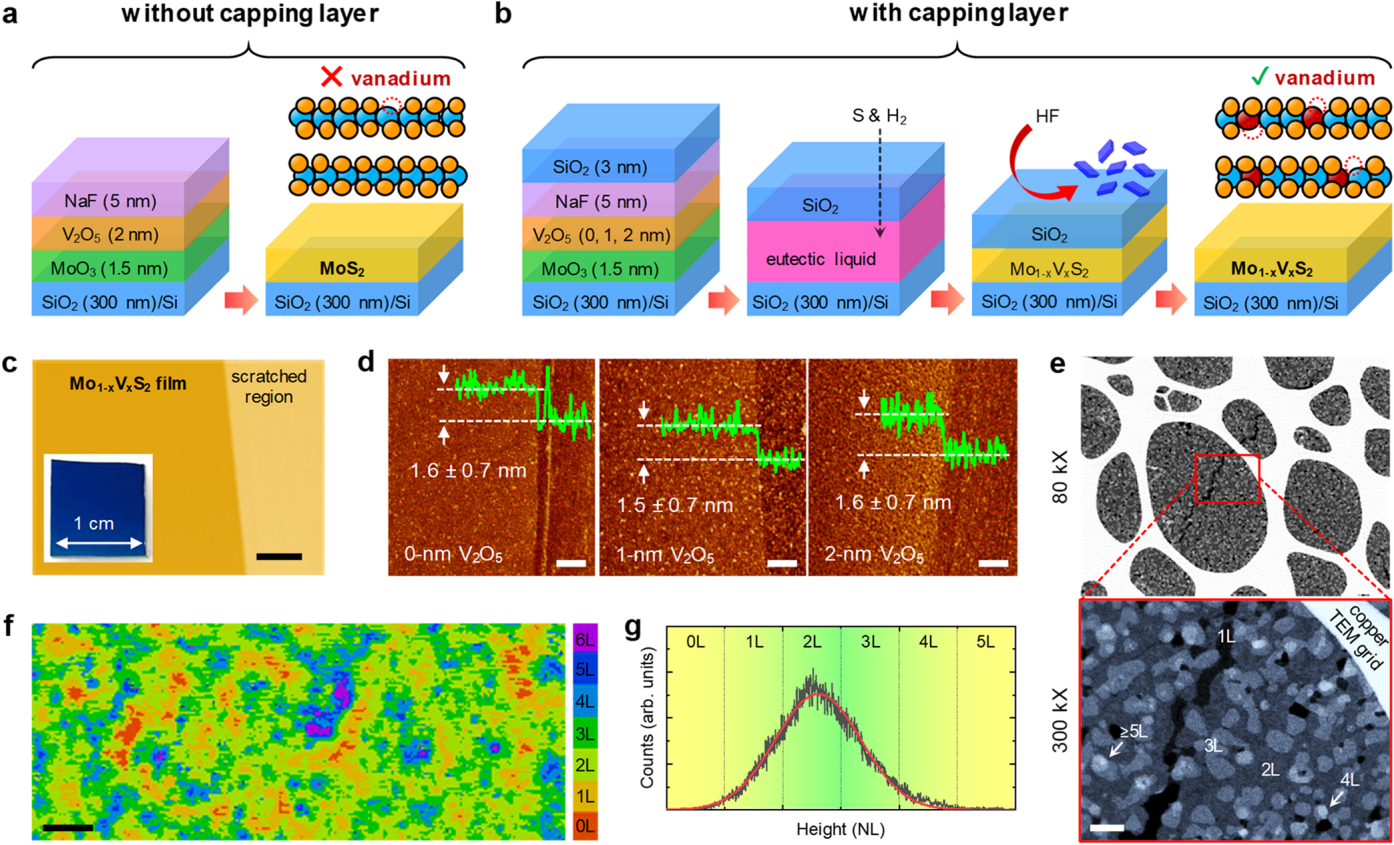
The membrane-controlled VLS growth schematics and topography of S_vac_-Mo_1–_ *_x_* V*_x_* S_2_ films. (a, b) Geometry of the solid precursors and the growth steps with and without a membrane, respectively. Blue-, yellow-, and wine-filled circles stand for Mo, S, and V atoms, respectively. (c) Optical microscopy image of the grown film. Scale bar: 20 μm. The inset displays a typical photograph of a 1 × 1 cm^2^ sample. (d) Wide scan atomic force microscopy (AFM) images of the grown film without the V_2_O_5_ layer (corresponding to pristine MoS_2_) and with 1 and 2 nm V_2_O_5_ layers (corresponding to Mo_1–*x*_V_*x*_S_2_). Scale bar: 1 μm. The insets show the height profiles. (e) Cs-corrected annular dark-field scanning transmission electron microscopy (ADF-STEM) images at different magnifications. Scale bar = 50 nm. (f, g) Zoomed-in AFM image and corresponding height profile histogram, respectively. Scale bar: 100 nm. The red curve shows the fitted Gaussian curve with a coefficient of determination of 0.98.

Since the formation of vanadium fluoride compounds is unavoidable, we proposed a membrane-controlled VLS growth method by depositing an ultrathin SiO_2_ capping layer on the solid precursors (Figure 1b). Concurrently, we optimized the thickness of the SiO_2_ membrane, the thickness of the MoO_3_ layer, the growth temperature, and the thickness of the V_2_O_5_ layer. Notably, optimization of the first three parameters was conducted for membrane-controlled VLS growth without using the V_2_O_5_ layer, *i.e.*, for the growth of pristine MoS_2_. (i) Thickness of SiO_2_ membrane: Figure S5 shows that the thick SiO_2_ membrane (or SiO_2_ capping layer) suppresses the diffusion of sulfur/hydrogen, leaving a large amount of the MoO_*x*_ phase. We observed that a membrane with a thickness of 3 nm can prevent the escape of vanadium and provide enough sulfur/hydrogen to react with an eutectic liquid via gas-in-solid diffusion. (ii) Thickness of the MoO_3_ layer: Figure S6 displays that the thickness of the film can be controlled by the thickness of the MoO_3_ solid precursor. (iii) Growth temperature: Our observation reveals that a growth temperature of 750 °C is the optimum temperature to grow a uniform and relatively high-quality film (Figures S7 and S8). For instance, it is well-known that the level of the disorder can shift E_2g_^1^ and A_1g_ vibrational modes in opposite directions and activate the phonon modes at the zone edge of the Brillouin zone, resulting in a larger frequency difference ω­(A_1g_) – ω­(E_2g_^1^) and smaller E_2g_^1^/LO­(M) intensity ratio (Figure S9).^44^ Moreover, atomic force microscopy (AFM) images show that the presence of large particles and rough surfaces for the films grown at temperatures ≥ 800 °C might be assigned to the upward diffusion of the eutectic liquid over the SiO_2_ membrane layer (Figure S10). (iv) Thickness of the V_2_O_5_ layer: As shown in Figure 1b, we deposited 1 and 2 nm V_2_O_5_ layer on the MoO_3_ layer. Optical microscopy and AFM images demonstrate the growth of full-coverage and wafer-scale film with an average thickness of 1.5 ± 0.7 nm (∼2L), where *x* depends on the thickness of the V_2_O_5_ layer (Figure 1c,d). As shown in Figure 1e, the Cs-corrected annular dark-field scanning transmission electron microscopy (ADF-STEM) images display that the film consists of interconnected (sub)­micron-size layers (the high-resolution images of microstructures of these 2L regions will be discussed later). Moreover, each layer contains disconnected thicker grains ≥ 3L (gray areas) with a characteristic size of <50 nm. Motivated by the observation of the variation in height profile, we recorded a zoomed-in AFM image with a slower scan speed and higher resolution of 1024 × 1024 (Figure 1f,g). It displayed that the films, consisting of interconnected grains in agreement with the ADF-STEM images, have a coverage of ≥97% and a Gaussian probability distribution for the height profile with the weights of *P*_i_ ≈ 16, 46, 29, 8, and 1% for NL_i_ = 1, 2, 3, 4, and ≥5L, respectively. Weighted arithmetic average results in a thickness of 2.3L, well-consistent with the wide-range-scan AFM images. Moreover, a thicker (≥3 nm) V_2_O_5_ layer led to a rough surface, possibly due to the high vapor pressure of vanadium fluoride gas and the collapse or break of the ultrathin membrane layer (Figures S11a,b and S12). However, it is worth noting that the Raman peaks associated with film grown with a thicker V_2_O_5_ layer are consistent with those observed in other vanadium-incorporated films (Figure S11c). The above-mentioned statements uncover that there should be a balance among the diffusion rate of sulfur/hydrogen into the SiO_2_ membrane layer, the concentration of vanadium in the eutectic liquid, and withholding the upward diffusion of the eutectic liquid over the membrane layer to grow uniform 2D alloy films.

### Characterizations of Ultrathin Films

We recorded the XPS spectra to identify the oxidation states, *x* values, and S_vac_ concentrations in S_vac_-Mo_1–*x*_V_*x*_S_2_ (Figure 2a). The fitted lineshapes show the peak positions at ∼516.7 eV (for V 2*p*_3/2_), 229.2–228.9 eV (for Mo 3*d*_5/2_), 226.4–225.9 eV (for S 2*s*), and 162.1–161.7 eV (for S 2*p*_3/2_), representing Mo^4+^, S^2–^, and V^4+^ oxidation states.^45,46^ These spectra display redshifts of 0.6 and 0.3 eV in the sulfur (first nearest neighbor) and molybdenum (second nearest neighbor) core-level binding energies assigned to the charge density transfer from low-electronegativity vanadium to the relatively high-electronegativity sulfur and molybdenum atoms, respectively. Utilizing the peak areas and dividing them by the corresponding atomic sensitivity factors (RSFs), the *x* = V/(V + Mo) ratio and S_vac_ concentration are calculated (Figure 2b). It shows *x* values of 0.18 and 0.30 when the V_2_O_5_ layer thickness is 1 and 2 nm, respectively. Results further indicate the presence of 5.7% S_vac_ in the pristine MoS_2_, which is the most commonly observed intrinsic point defect in the TMDCs due to its low formation energy,^47,48^ to 16.2% S_vac_ in Mo_0.7_V_0.3_S_2_. Notably, we observed 17 and 35% broadening in the full width at half-maximum (FWHM) of the Mo 3*d*_5/2_ and S 2*p*_3/2_ lineshapes, respectively, which can be assigned to the change of the local chemical environment after vanadium incorporation and the concurrent presence of S_vac_. As shown in Figures 2c and S13, Bader charge analysis displays significant charge density transfer to sulfur and molybdenum atoms after the introduction of vanadium and S_vac_, consistent with the XPS results. This suggests that the nearest neighbor sulfur and molybdenum atoms have a relatively greater electron density. Moreover, our density functional theory (DFT) calculations showed the average S_vac_ formation energies of 2.67 (2.69) to 2.20 eV (1.21 eV) for the 2L (1L) films with *x* = 0 and 0.3, respectively (Figure S14). In agreement with the XPS results, is a correlation between vanadium and S_vac_ concentrations, *i.e.*, stabilization of S_vac_ via substitution of molybdenum with vanadium. Formation energy calculations for different systems also indicate a tendency for S_vac_ to be located near vanadium atoms.

**Figure 2:**
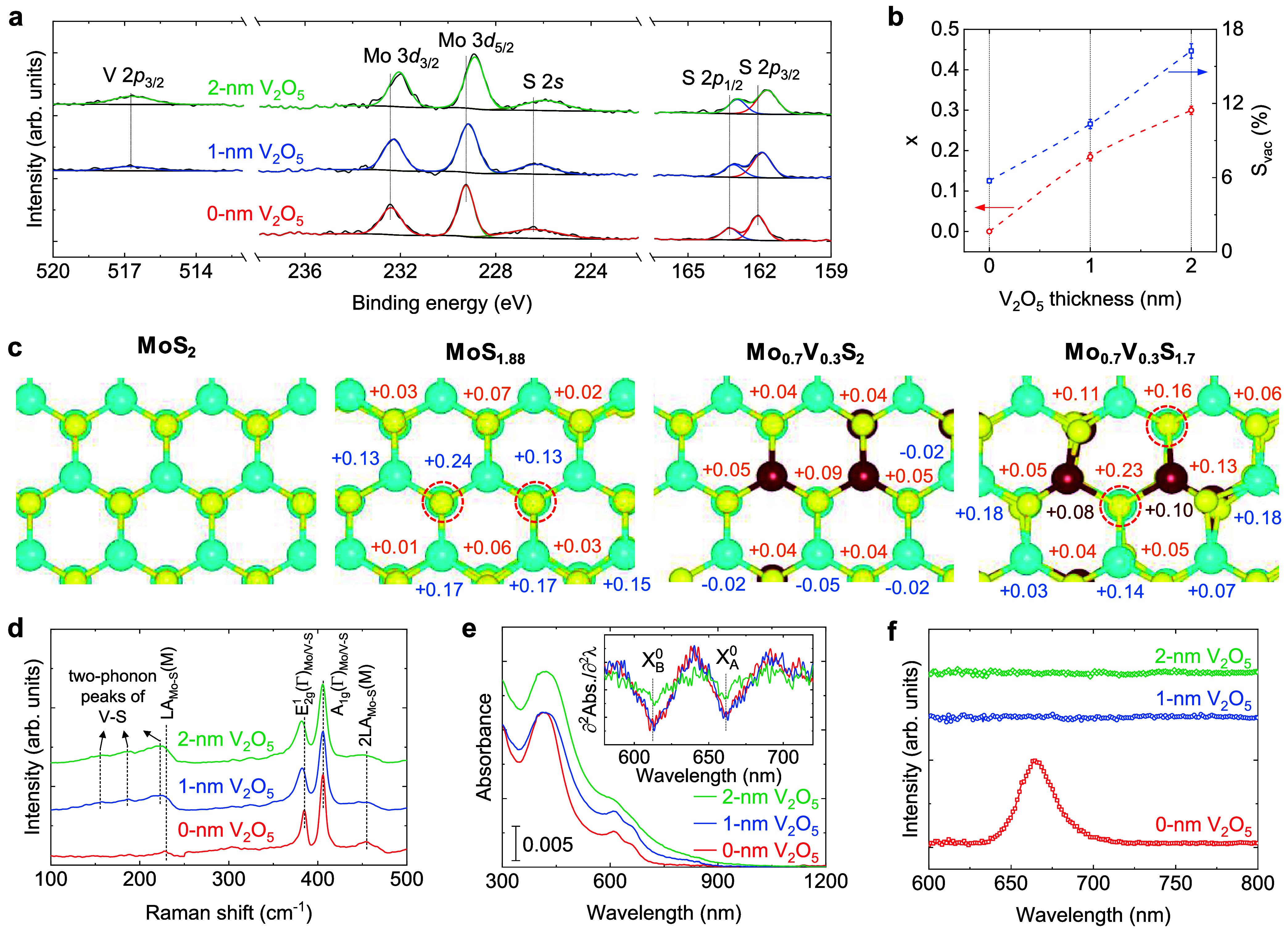
Characterization of the grown films. (a) XPS spectra of V 2*p*, Mo 3*d*, and S 2*p*. (b) *x* and S_vac_ versus the V_2_O_5_ layer thickness. (c) The DFT relaxed configurations and the surface Bader charge (valence electron) differences compared to the pristine MoS_2_ of the 2L Mo_1–*x*_V_*x*_S_2_. blue-, yellow-, and wine-filled circles stand for Mo, S, and V atoms, respectively. The presence of vanadium and S_vac_ slightly distorts the hexagonal crystal structure. (d–f) Raman spectra, absorbance curves, and photoluminescence (PL) spectra, respectively. The inset of panel (e) plots the second derivative of absorbance with respect to wavelength (λ). Notably, PL experiments for the vanadium-containing samples do not show any signal for λ < 800 nm.

The lattice vibrational modes were investigated by Raman spectroscopy (Figure 2d and Table S1). The Raman spectrum of MoS_2_ shows two prominent peaks at 384.2 and 405.7 cm^–1^ and also two small peaks at 227.4 and 453.5 cm^–1^ corresponding to the in-plane E_2g_^1^(Γ)_Mo–S_, out-of-plane A_1g_(Γ)_Mo–S_, longitudinal acoustic LA­(M)_Mo–S_, and 2LA­(M)_Mo–S_ vibrational modes, respectively. After the introduction of vanadium, the A_1g_(Γ)_Mo–S_ mode remains almost invariant of 405.5 cm^–1^, and the E_2g_^1^(Γ)_Mo/V–S_ mode redshifts to 380.6 cm^–1^ and exhibits a larger FWHM assigned to the substitution of molybdenum with vanadium and short-range structural disorder in the hexagonal crystal structure (see Table S1 and Figures 2c and S14a), respectively. In addition, alloyed films display several peaks in the range from 100 to 250 cm^–1^ due to the two-phonon processes, which are related to the V–S vibrations.^33,46^ As shown in Figure 2e, absorbance curves depict a significant redshift in the absorption onset and achieve more light absorption by increasing *x*, although the wavelengths of the direct KK transitions,^49^ *i.e.*, neutral excitons *X*_A_^0^ at ∼662 nm (∼1.87 eV) and *X*_B_^0^ at 612 nm (∼2.02 eV),^50^ are invariant. Therefore, vanadium incorporation results in higher light harvesting with an overall absorption of β_AM 1.5G_ ≈ 4.0% (Figure S15). Notably, the exponential behavior of the absorption coefficient at long wavelengths (known as Urbach tail) can be allocated to the presence of midgap defect states, which is confirmed by our DFT calculations (Figures S16 and S17).^51^ The photoluminescence (PL) spectroscopy was used to study the radiative recombination (Figure 2f). The PL spectrum of the pristine MoS_2_ shows an asymmetric peak attributed to the presence of exciton or trion quasi-particles and also thickness variation of about ±1L. We further observed that the PL signals are completely quenched by increasing *x*. The PL quenching and extended absorption tail can be attributed to the existence of abundant midgap states (boosting the nonradiative recombination rate) originating from the presence of vanadium and S_vac_.

### Microstructures and Stacking Orders

We conducted ADF-STEM imaging to explore the atomic arrangements, vacancies, morphology, crystal structure distortion, grain boundaries, and stacking orders (Figures 3, S18, and S19). It is worth noting that by assuming the *Z*-contrast of each element is proportional to *Z* ^1.7^ where *Z* is the atomic number,^52,53^ we can approximately differentiate between sulfur, vanadium, and molybdenum atoms as well as vacancies. When imaging each column from the vertical direction of the 1L hexagonal TMDCs, which consist of either one metal or two chalcogen atoms, the intensity ratio is approximately *I*_Mo_/*I*_2S_/*I*_V_ ≈ 5:2:2. Nevertheless, the relative difference in brightness between the cation and anion columns displays a hexagonal crystal structure.^54^ We have further observed that the *Z*-contrasts of the columns in the thicker regions diminish, especially those not twisted, making it challenging to precisely differentiate between atoms or vacancies. For example, each sulfur column in 2L or 3L MoS_2_ with AA/AAA stacking orders contains four or six sulfur atoms. The presence of S_vac_ leads to about a 10% and 6% reduction in brightness compared with the molybdenum column, respectively, as determined by elastic-screened Rutherford scattering.

**Figure 3:**
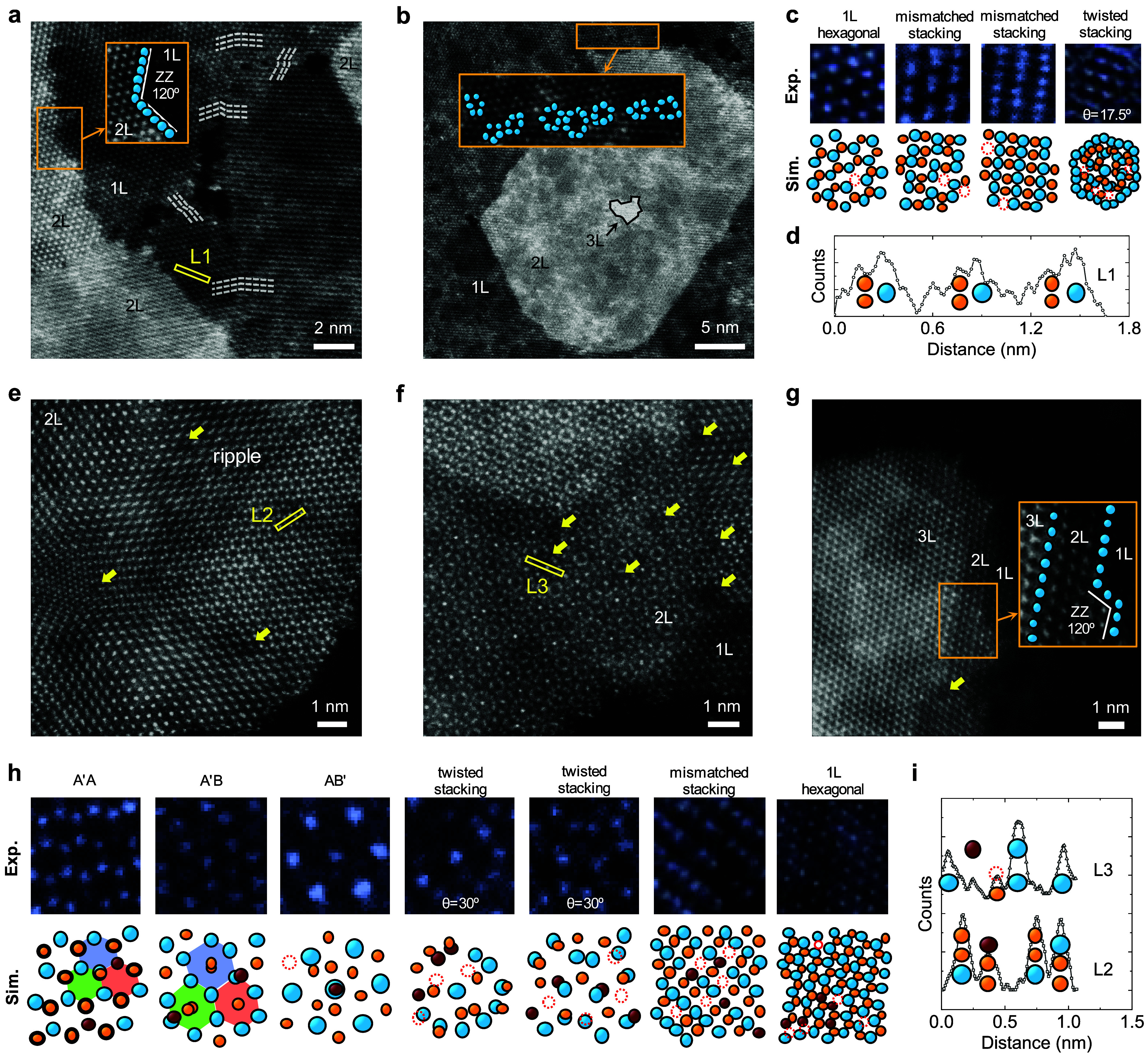
Microstructure and stacking order of the grown films. (a–c) ADF-STEM images, zoomed-in images, and corresponding arrangements of the elements of the MoS_2_ film from different regions. (d) Intensity profiles along the corresponding line (L1) in (a). (e–h) ADF-STEM images, zoomed-in images, and corresponding arrangements of the elements of the Mo_0.7_V_0.3_S_2_ film from different regions. The yellow arrows indicate regions with higher concentrations of vanadium. (i) Intensity profiles along the corresponding lines (L2 and L3) in (e, f). Blue, yellow, wine-filled, and red circles stand for Mo, S, V atoms, and S_vac_, respectively. Notably, the ADF-STEM images were Gaussian blurred with a sigma value of 2. A′A, A′B, and AB′ represent three different local stacking orders.

Atomic-resolution STEM images show that each grain consists of boundaries where the crystal orientations of the TMDC layers on both sides are different, suggesting the connection area between the flakes. We have also noticed the existence of thicker regions, mainly dominated by 2L layers with zigzag (ZZ) terminated edges. It is important to note that while the thicker regions exhibit preferable edge characteristics, their shape does not resemble the expected triangular structure. As a rule of thumb, it can be stated that their shape mostly bears a likeness to an elongated sphere (quasisphere) in low magnification. The presence of these regions, along with their shape, indicates that they form during the final step of growth when the liquid droplet is reduced in size. The same phenomenon was observed by the VLS epitaxial-grown MoS_2_ ribbons, named the island of multilayered MoS_2_.^29^ In addition, STEM images unveil different stacking orders (such as mismatched, twisted, A′A, A′B, and AB′ stacking orders) and ripples in ≥2L regions due to the presence of strain and local separation of the layers.^29,55^ These features are primarily a result of the stress induced by liquid crawling during the growth process. High-resolution STEM images reveal a relatively higher density of S_vac_ and slight distortion in the hexagonal crystal structure after vanadium substitution into molybdenum sites, aligning with our earlier discussed experimental and theoretical results. At the end, the 2L film exhibits some low-brightness regions due to the presence of nearby vanadium atoms and S_vac_ sites, which we will discuss in the following text by simulation of the atomic configuration.

### Simulation of Atomic Configuration

We proposed a model for S_vac_-Mo_1–*x*_V_*x*_S_2_ with 16.2% S_vac_ and *x* = 0.3 to evaluate the probability distribution of the number of nearest neighbor vanadium atoms (*N*_NN_^V^) and S_vac_ sites (*N*_NN_^S_vac_^) around each vanadium atom, named *P*_V–V_(*N*_NN_^V^) and *P*_V–S_vac__(*N*_NN_^S_vac_^), respectively. We considered two types of systems to model the atomic configuration. The first one is the random dispersion of atoms, and the second one is the biased dispersion with a free parameter α_VV_ from 1 to 100%. The smaller α_VV_ indicates a lower probability of vanadium atoms being far from each other. As shown in Figure 4a,b, the transparent green and purple shapes illustrate that the biased dispersion contains fewer isolated V atoms and S_vac_ sites, implying a larger fraction of V–S_vac_ pairs. To quantify this observation, we calculated *P*_V–V_ and *P*_V–S_vac__ versus *N*_NN_^V^ and *N*_NN_^S_vac_^, respectively. Figure 4c displays that the high-probability *N*_NN_^V^, where *P*_V–V_ reaches its maximum, increases from 1.63 to 2.62 for the random and biased dispersions, respectively. Moreover, the probability of vanadium being isolated from other vanadium atoms, *i.e.*, *P*_V–V_(*N*_NN_^V^ = 0), decreases by more than two times. Further, the probability distribution of the number of nearest neighbor S_vac_ sites around each vanadium atom shows the same behavior (Figure 4d). For instance, the high-probability *N*_NN_^S_vac_^ increases from 0.65 to 1.16 and *P*_V–S_vac__ (*N*_NN_^S_vac_^ = 0) decreases from 0.35 to 0.22 for the random and biased dispersions, respectively. These plots indicate that considering the DFT-calculated formation energies of vanadium and S_vac_ within the host MoS_2_ crystal structure results in the formation of V–S_vac_ pairs, with approximately 70% (for α_VV_ = 100%) to 82% (for α_VV_ = 1%) of vanadium atoms paired with S_vac_ sites. This suggests that the observed behavior is a likely occurrence within this system from a thermodynamic perspective. However, it is important to note that the presence of an S-rich atmosphere during the growth process, as well as elevated growth temperatures or longer reaction time, could influence the probability of V–S_vac_ pair formation, indicating a need for further investigation in this area.

**Figure 4:**
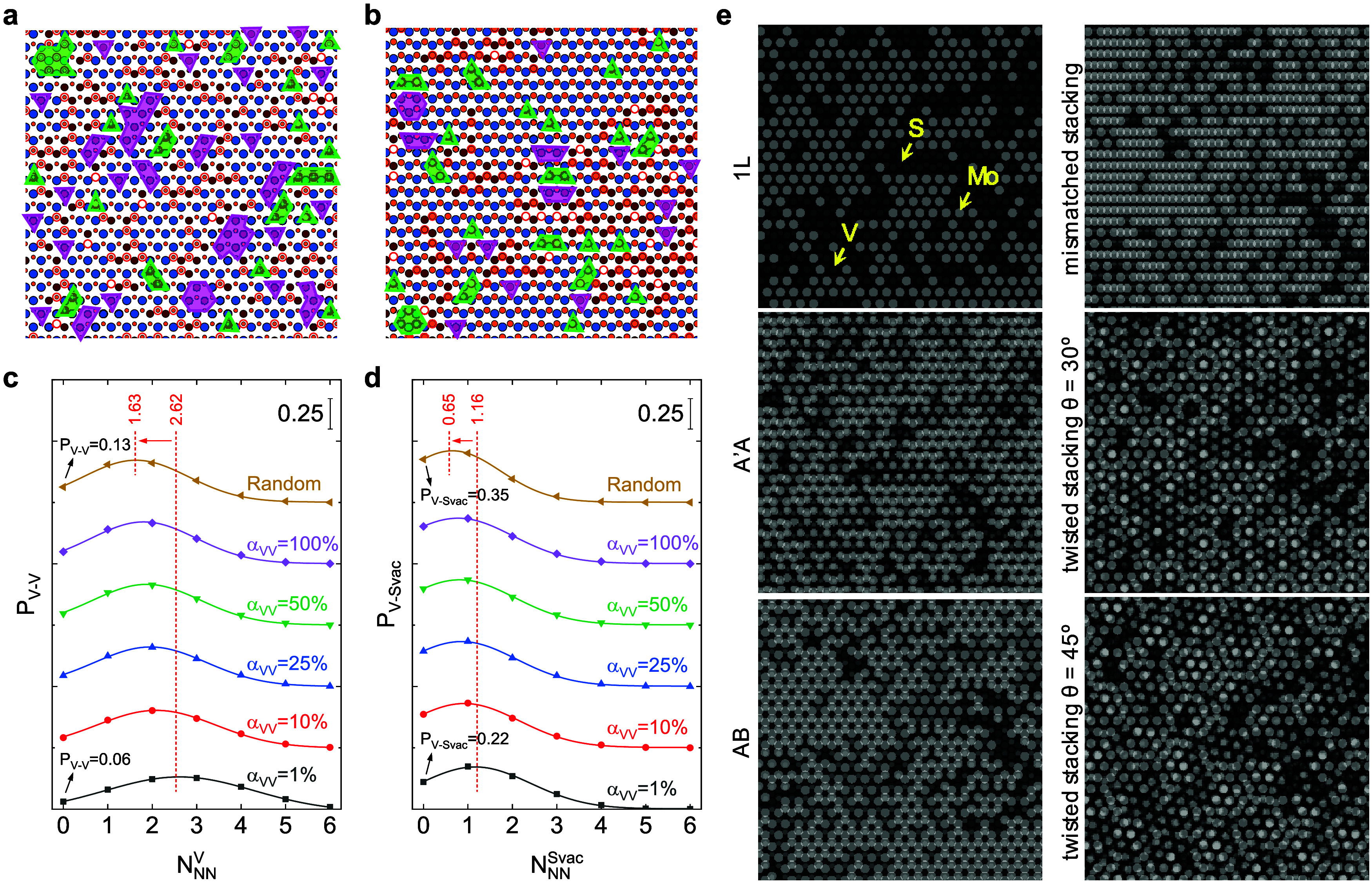
Modeling of atomic configuration. (a, b) Random and biased (α_VV_ = 1%) dispersions of atoms, respectively. Blue, yellow, wine-filled, and red circles represent Mo, S, and V atoms and S_vac_, respectively. The transparent green shapes indicate S_vac_ sites without any nearby vanadium atoms, while the transparent purple shapes indicate vanadium atoms without any nearby S_vac_ sites. (c, d) *P*_V–V_ and *P*_V–Svac_ as a function of *N*_NN_^V^ and *N*_NN_^Svac^, respectively, for biased dispersion with different α_VV_ percentages and random dispersion. Notably, a Gaussian function is used to fit all of the data points. (e) Modeled STEM images of 1L and 2L S_vac_-Mo_1–*x*_V_*x*_S_2_ with different stacking orders and twisted angles. All data were generated for 16.2% S_vac_ and *x* = 0.3.

Our model is also used to simulate STEM images by considering that the *Z*-contrast is proportional to *Z* ^1.7^. Figure 4e presents several examples of 1L and 2L S_vac_-Mo_1–*x*_V_*x*_S_2_. Although the *Z*-contrast of the atomic columns in the 2L film is lower than in the 1L film, making it somewhat challenging to assign all the atoms, there are several low-brightness regions caused by nearby vanadium atoms and S_vac_ sites, consistent with our recorded ADF-STEM images (e.g., see the yellow arrows in Figure 3).

### Membrane-Controlled VLS Growth Mechanism

Here, we provide an overview of our qualitative comprehension of the growth mechanisms. Our insights stem from the experimental observations and relevant previous reports.^29,30^ Figure S20a,b illustrates the formation of the eutectic liquid through the reaction involving MoO_3_, V_2_O_5_, and NaF. Subsequently, hydrogen and sulfur dissolve into the liquid via gas-in-solid diffusion until it reach saturation, thereby triggering the formation of the first nucleations at the droplet–substrate interface. The continuous precipitation of Mo_*x*_V_1–*x*_S_2_ in the ultraconfined space consumes the liquid and induces bubble formation due to reaction products such as gas-phase molybdenum difluoride dioxide, water, and molybdenum vanadium fluoride. The last low-boiling point compound is proposed because of significant vanadium-dependent molybdenum losses during growth processes (Figure S21). Once the film grows, the liquid crawls back to minimize the total interfacial free energy, Δ*G*_int_ = (σ_LF_ – σ_LS_)*A*_LF_. The 2D circular symmetry due to this specific geometry implies that the film should grow in all directions, resulting in the growth of quasi-spherical grains. The radial growth, caused by the liquid back-crawling, explains the presence of grain boundaries with distinct crystal orientations on both sides. The observation of different stacking orders and ripples may be attributed to the compressive stress induced by the liquid back-crawling. This process continues until the bubble reaches the SiO_2_ membrane layer, triggering the emergence of a vertical capillary force due to the liquid curvature. As shown in Figure S20c, such a capillary force generates tensile stress on the SiO_2_ membrane layer and would result in the formation of cracks right after the growth process. The AFM image before etching the membrane layer displays the presence of cracks thereon, confirming our proposed mechanism (Figure S20d,e). In addition, the residual liquid droplet on the 2L film results in the precipitation of multilayered islands.

To gain insights into the influence of the utilized salt, we investigated the properties of the films by varying the thickness of the NaF solid layer from 2 to 11 nm. As illustrated in Figure S22, increasing the thickness of NaF leads to a higher number of nucleation sites and results in a rougher surface due to the tendency for vertical growth. Additionally, we examined other salts, specifically 5 nm NaCl, which also produced significantly higher roughness (Figure S23). It is important to note that the use of alternative salts would require a separate investigation focused on optimizing the sulfurization temperature, pressure, growth time, and even thickness of solid precursors.

### Photocatalytic Water-Humidified CO_2_ Reduction

The gas-phase photocatalytic (PC) experiments were conducted in a water-humidified CO_2_ environment, resulting in a selective and stable CO product for five cycles (or 20 h) (Figures 5a–c and S24).^56^ Our experiments demonstrate that the presence of S_vac_ enhances the PC activity,^8^ which is further boosted after the substitution of vanadium into molybdenum sites. The observed enhancement can be attributed to several factors such as better light absorption, reaction kinetics for the actual interaction between reactants and surface-active sites, altered energy band positions, CO_2_/H_2_O adsorption on the active sites, and enhanced charge transfer/transport.^57^ The first factor was substantiated earlier by measuring the overall light absorption of S_vac_-Mo_1–*x*_V_*x*_S_2_, which is ∼1.7 times larger than pristine MoS_2_. To explore the third factor, we conducted light-intensity-dependent photocatalytic experiments. Figure 5d illustrates that the CO yield of the optimized film is inversely proportional to the light intensity, reaching a maximum internal quantum efficiency (IQE) of 0.017% at 0.5 sun. This implies that light absorption is not the rate-determining step, and the reduction and oxidation reaction rates are different.^58^ Given that the relative positions of the conduction and valence bands present upward shifts, the third factor plays a positive role due to the varying reduction and oxidation driving forces (Figures S25, S26, and 5e). To address CO_2_/H_2_O adsorption on the active sites, we performed DFT simulations to calculate the binding energies of reactants on the surface of the model photocatalyst with S_vac_ as a function of vanadium concentration,^6,17,59^ revealing that the introduction of vanadium significantly aids in the absorption of H_2_O but not CO_2_ molecules (Figure 5f,g). Calculations indicate that S_vac_ in the vicinity of vanadium atoms can further regulate the binding energy at the transition state (Figure 5h). DFT simulations suggest that H_2_O and CO_2_ molecules tend to absorb the exposed vanadium atoms, indicating that the V–S_vac_ pairs are the active sites. Using the methodology introduced by Qorbani et al.,^12^ we estimated that transferred electron rates per active site *R*_CO_^S_vac_^ ≈ 0.005 and 0.010 e^–^ s^–1^ with and without vanadium, respectively, highlighting the role of the V–S_vac_ pairs in activating the basal plane of MoS_2_.

**Figure 5:**
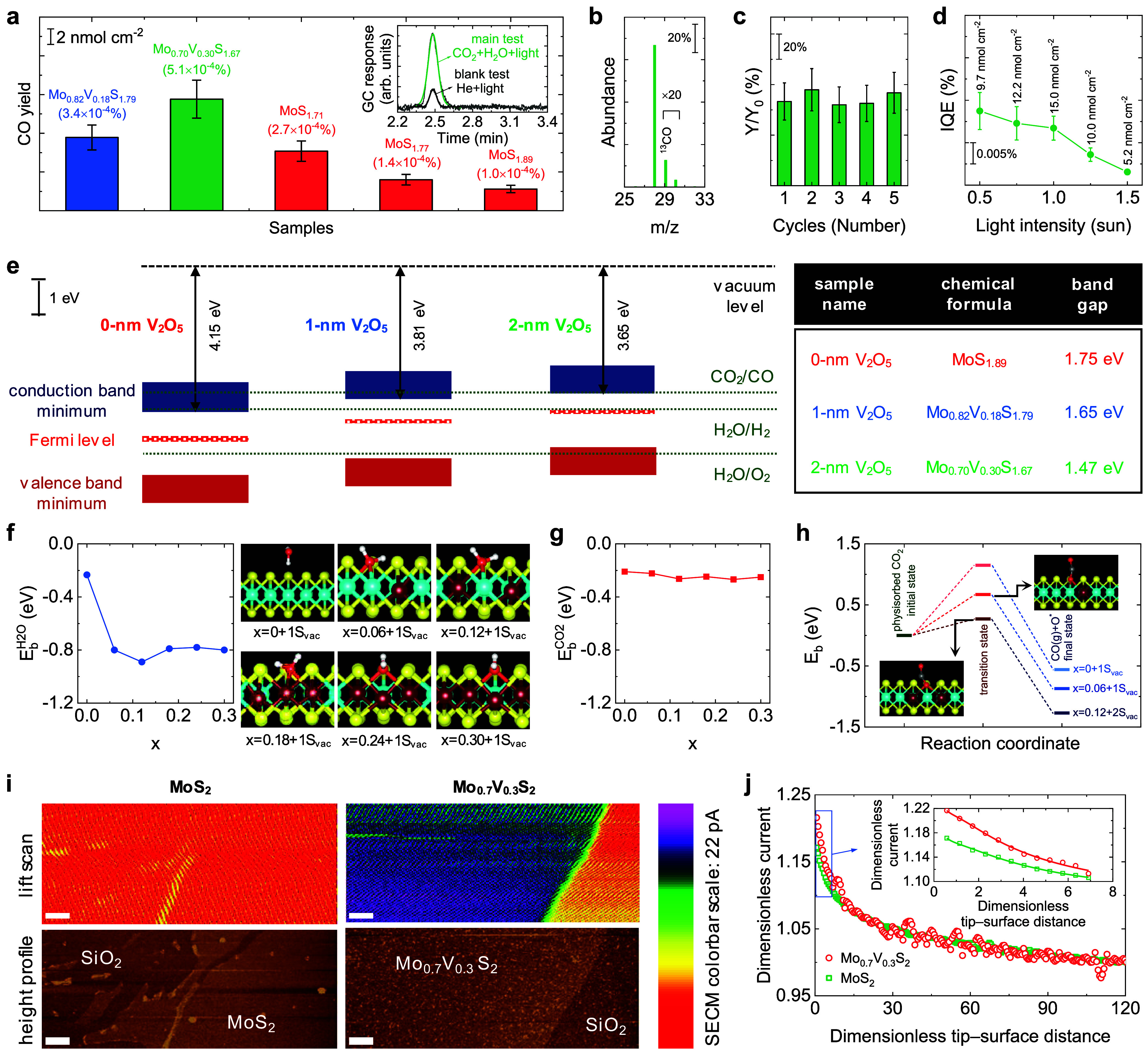
Photocatalytic performance. (a) Blank-corrected total CO yield after light irradiation for 4 h. The inset shows the gas chromatography (GC) response for the main test in CO_2_ + H_2_O and the background test in He environments under light irradiation. Moreover, the apparent quantum efficiency (AQE) of each PC test is written in each column. (b) Gas chromatography–mass spectrometry (GC–MS) of the isotope tracer ^13^CO_2_ measurement. The peaks at *m*/*z* = 28 and 29 are assigned to N_2_ and ^13^CO, confirming that the PC reaction is the only source of the CO product. (c) Stability test for five cycles. Irradiation time for each cycle is 4 h. (d) IQE vs light intensity. The product yield of each PC test is written in each column. (e) Experimental energy band diagram. The position of Fermi (or chemical potentials), H_2_O/O_2_ oxidation, H_2_O/H_2_ reduction, CO_2_/CO reduction levels, and band gap are also depicted as the reference. (f) Binding energy of H_2_O (*E*_b_^H2O^) versus *x* (in S_vac_-Mo_1–*x*_V_*x*_S_2_) with 1S_vac_ and corresponding and calculation-based relaxed configurations of H_2_O molecules on the surface. (g) Binding energy of CO_2_ (*E*_b_^CO2^) versus *x* with 1S_vac_. (h) DFT calculation results of the chemical reaction pathway for CO_2_–to–CO reduction. The insets show the transition states for *x* = 0.06 with 1S_vac_ and *x* = 0.12 with 2S_vac_. Blue-, yellow-, wine-filled, red, white, and silver circles stand for Mo, S, V, O, H, and C atoms. (i) AFM height profile measured in the liquid environment and SECM feedback maps for lift scans. Scale bar = 2 μm. (j) SECM normalized dimensionless response as a function of the dimensionless tip–surface distance for pristine MoS_2_ and S_vac_-Mo_1–*x*_V_*x*_S_2_. The inset displays the zoomed-in SECM normalized response. The lines represent the fit with an analytical approximation.

To investigate the charge transfer and transport properties, we collected the AFM-based SECM feedback maps for lift scans. In this approach, the probe is elevated to a predetermined and constant height (100 nm) above the surface while precisely adhering to the previously recorded topographic profile from the main scan, where it operates close to the surface. Consequently, the electrochemical current, or SECM signal, is recorded as a function of the lateral position. By maintaining a constant tip–sample distance throughout the lift scan, the observed current variations can be attributed exclusively to localized changes in the surface’s electrochemical activity. As shown in Figure 5i, the positive normalized dimensionless feedback current maps illustrate that the local electrochemical activity of S_vac_-Mo_1–*x*_V_*x*_S_2_ is an order of magnitude larger than pristine MoS_2_, *i.e.*, the SECM current of the pristine MoS_2_ and S_vac_-Mo_1–*x*_V_*x*_S_2_ is ≤1 and ∼15 pA, respectively. The local electrochemical activity can be attributed to charge transfer at the interface and transport in the basal plane. This result is in agreement with the DFT calculations, where we observed a better interaction between the water molecule and the surface and also an enriched DOS of S_vac_-Mo_1–*x*_V_*x*_S_2_. A recent report shows that an elevated electronic DOS near the Fermi level significantly enhances the rate of interfacial electron transfer.^60^ This increase in DOS promotes a higher probability of out-of-plane electron tunneling at the electrode–electrolyte interface, consequently facilitating improved in-plane charge transport. Figure 5j not only confirms the SECM feedback current maps but also shows that the normalized dimensionless response as a function of the dimensionless tip–surface distance was well fitted with an analytical approximation.

## Conclusions

In summary, we have developed a capped (membrane-controlled) vapor–liquid–solid growth method to synthesize wafer-scale and ultrathin S_vac_-Mo_1–*x*_V_*x*_S_2_ alloyed films. By controlling the thickness of solid precursor MoO_3_ and V_2_O_5_ layers, the SiO_2_ membrane, and the growth temperature, the thickness and vanadium concentration have been precisely controlled. We further unveiled that vanadium atoms can stabilize S_vac_ sites, resulting in the formation of V–S_vac_ pairs. The presence of V–S_vac_ pairs emerged in a relation between vanadium and S_vac_ concentrations, charge transfer among the V–S–Mo atoms, better light absorption due to enriched DOS, photoluminescence quenching, the ability to bind reactant CO_2_ and H_2_O molecules during the PC experiments, and efficient charge transfer/transport observed from a nanoscale redox mapping. Our proposed model, based on input from the DFT simulations, offered further insights into the distribution of the alloying elements and S_vac_ sites. In addition, as a model photocatalyst for gas-phase CO_2_ reduction with water vapor, S_vac_-Mo_1–*x*_V_*x*_S_2_ significantly enhanced the CO_2_ to CO conversion efficiency mainly due to the presence of V–S_vac_ pair active sites.

Broadly, we believe that the introduced capped VLS growth method can be realized for other 2D materials beyond MoS_2_ and dopants/alloying elements beyond vanadium by identifying a suitable liquid-phase intermediate compound. Thus, our findings offer insights into novel synthesis approaches for 2D materials and prospects for the development of unique multielement dopants/alloying. In addition to the growth method, the realization of an alloying element–defect pair active site opens a door for further studies in the field of solar fuel, such as photocatalytic, photoelectrochemical, or even electrochemical carbon dioxide reduction and water splitting applications.

## Experimental Methods

### Preparation of Solid Precursors

A 1 × 1 cm^2^ SiO_2_(300 nm)/Si substrate was initially cleaned with deionized water and subsequently sonicated in acetone and isopropyl alcohol. MoO_3_ films with various thicknesses (1.5–4 nm) were grown on top of the substrates with PEALD using Mo­(CO)_6_ (purity >98%, supplier: Alfa Aesar) and oxygen plasma as the precursor and oxidation reactant, respectively. The chamber pressure and temperature of the substrate were 0.18 Torr and 200 °C during the PEALD process, respectively. Various thicknesses of V_2_O_5_ (from 1 to 7 nm), NaF (from 2 to 11 nm), and SiO_2_ (3 and 6 nm) films were deposited by EBE using V_2_O_5_ (purity >99.95%, supplier: ELECMAT), NaF (purity >99.99%, supplier: ELECMAT), and SiO_2_ (99.99%, supplier: ELECMAT) pieces with a chamber pressure of 8 × 10^–6^ Torr. For the EBE, film thickness was monitored using a quartz crystal microbalance, and the deposition rate was maintained at 0.1 Å s^–1^. Substrates were affixed to a spinning sample holder to ensure high uniformity. Nevertheless, the optimized configurations of the solid precursors on the insulating substrate were SiO_2_(3 nm)/NaF­(5 nm)/MoO_3_(2 nm)/V_2_O_5_(0–2 nm)/SiO_2_(300 nm)/Si. S_vac_-MoS_2_ films were prepared by thermal annealing of MoS_2_ films at 300 °C and chamber pressure of 1.0 Torr for 20 and 40 min, with a fixed flow rate of 10 and 90 standard cm^3^ min^–1^ for H_2_ and Ar carrier gases, respectively.^61^

### Growth of Ultrathin Films

The sulfurization process was conducted within a 2 in. quartz tube, utilizing a low-pressure three-zone horizontal CVD system. 300 mg sulfur powder (purity >99.98%, supplier: Sigma-Aldrich) and solid precursors were placed in an alumina crucible and on a 3 × 3 cm^2^ quartz plate at zone-1 and zone-3, respectively. The temperature of the sample was incrementally raised at a rate of 40 °C min^–1^, reaching the desired temperature (from 450 to 900 °C), and maintained at this temperature for a duration of 10 min. It should be noted that the optimized growth temperature was obtained at 750 °C. Sulfur vapor was introduced by gradually increasing the temperature at a rate of 15 °C min^–1^, reaching 145 °C, and then maintaining at this temperature for 10 min. The system’s pressure was upheld at 1 Torr, with a fixed flow rate of 0.5 and 50 standard cm^3^ min^–1^ for H_2_ and Ar carrier gases, respectively. Following sulfurization, the specimen was immersed in a diluted buffered oxide etch 5%(v/v)-HF solution for about 30 s to eliminate the SiO_2_ membrane layer.

### Measurements

Optical microscopy images were captured using an Olympus BX53 M microscope. The thickness and surface roughness of the films were assessed by both AFM from Bruker AXS in noncontact mode, employing an arrow-type silicon AFM probe with a diameter of less than 16 nm (NanoWorld; NCHR-50). The AFM operation was controlled by a feedback mechanism, with the cantilever driven at a resonance frequency of ∼330 kHz and a spring constant of 42 N m^–1^. XPS and ultraviolet photoelectron spectroscopy (UPS) spectra were collected by a VG Scientific ESCALAB 250 spectrometer and ULVAC PHI 5000 Versa Probe under an Al *K*_α_ (1486.6 eV) and He I (21.2 eV) radiation source. All samples were transferred onto the Au-coated SiO_2_/Si substrate for conducting UPS and XPS experiments by a wet-transfer method. XPS spectra were calibrated by the C 1*s* peak at 284.6 eV. Energy band diagrams were calibrated by the Au substrate work function at 5.1 eV. The CasaXPS software (version 2.3.20) was used to fit the XPS data using Shirley background and with Gaussian–Lorentzian product GL­(*m*), for C 1*s*, Mo 3*d*, and S 2*p* signals, and Gaussian–Lorentzian sum SGL­(*m*), for S 2*s* signal, lineshapes. We adjusted the *m* value in the mentioned lineshapes using the “Test Peak Model” in CasaXPS software.^62^ We have used the following equations to find the energy band diagram, *i.e.*,

1 $$\mathrm{WF}=h\nu -\left(\mathrm{SECO}+{\mathrm{eV}}_{\mathrm{bias}}\right){|}_{\mathrm{by}\;\mathrm{SECO}\;\mathrm{edge}}$$

2 $$\mathrm{VBM}=\mathrm{WF}-\left(\mathrm{WF}-\mathrm{VBM}\right){|}_{\mathrm{by}\;\mathrm{Femi}\;\mathrm{edge}}$$

and

3 $$\mathrm{CBM}=\mathrm{VBM}+E_{g}{|}_{\mathrm{by}\;\mathrm{UV}-\mathrm{visible}}$$

where WF, *h*ν = 21.2 eV, SECO, *V*_bias_ = 5 V, CBM, VBM, and *E*_g_ are work function, He I ionization energy, secondary electron cutoff (SECO), bias voltage, conduction band minimum, valence band maximum, and band gap, respectively. Energy band diagrams were calibrated by the Au substrate work function at 5.1 eV. It should be noted that measured Au substrate work functions are 4.1, 3.94, and 3.72 eV for the films without V_2_O_5_ (pristine MoS_2_) and with 1 and 2 nm V_2_O_5_ layer (Mo_1–*x*_V_*x*_S_2_), respectively. Transmission electron microscopy (TEM) and STEM imaging were conducted using the JEOL JEM-2100F and JEOL JEM-ARM300F2, operated at 100 and 80 kV, respectively. The STEM system is equipped with double spherical aberration correctors and energy-dispersive X-ray spectroscopy (JED EX34400MNU). The digital micrograph software package (GMS 3, Gatan Microscopy Suite 3) was used to analyze the STEM images. PL and Raman spectra were collected on confocal HORIBA (iHR550) systems using a green (532 nm) laser. UV–visible spectra were obtained using a double-beam spectrophotometer, specifically the Jasco V-670 model. The measurements were conducted on a film transferred onto a quartz substrate. The following equation was used to calculate the overall absorption percentage (β_AM 1.5G_)­

4 $$\beta_{\mathrm{AM}\;1.5G}=\frac{\int [1-10^{-A_{\theta }\left(\lambda <\lambda_{g}\right)/\theta }]\phi \left(\lambda \right)d\lambda }{\int \phi \left(\lambda \right)d\lambda }$$

where λ [nm], *T*(λ < λ_g_), θ, *A*_θ_(λ), and ϕ­(λ) [s^–1^ cm^–2^] are wavelength, coverage, coverage-dependent transmittance, and incident photon flux of the Xe lamp, respectively.^12^ Coverage is defined as the ratio of the area of the transferred film to the optical slit of the apparatus. Moreover, the poly­(methyl methacrylate) (PMMA)-assisted wet-transfer method was applied to transfer the as-grown ultrathin films. Thermogravimetric analyses were performed utilizing a thermal analysis instrument (TA Instrument Q500), ranging from room temperature up to 750 °C and within an atmosphere of Ar.

### DFT Simulations

Briefly, we employed spin-polarized DFT calculations, implemented in the Vienna ab initio Simulation Package (known as VASP).^63^ The electron–ion interaction was modeled using the projector augmented-wave method.^64^ The exchange-correlation functional was defined by the generalized gradient approximation within the Perdew–Burke–Ernzerhof (known as PBE) scheme, with the inclusion of the dispersion-correction DFT-D3 method.^65,66^ The electron wave functions were expanded using a plane-wave basis set with a kinetic energy cutoff set at 500 eV. Furthermore, a Hubbard on-site correction term, with a consistent *U*_eff_ = 3 eV, was specifically applied to the vanadium atoms.^67–69^ Throughout the geometry optimization, the convergence criteria for electronic and ionic iterations were set at 1 × 10^–5^ eV and 0.01 eV Å^–1^, respectively. In this study, supercells consisting of 4 × 4 monolayer and bilayer models of MoS_2_ were employed. A vacuum distance of 25 Å was established in the out-of-plane direction to mitigate interactions with the periodic images. The Brillouin zone was sampled utilizing γ-cantered *k*-point meshes, with a grid of 3 × 3 × 1 for geometry optimization and 6 × 6 × 1 for density of states calculations. Bader charge analysis, employing the weight method, was conducted to partition and integrate the electronic charge densities for each atom.^70,71^ We further calculated the frequency-dependent dielectric function to obtain the linear optical properties within the sunlight range.^72,73^ VESTA (3.5.7) software was used to draw the relaxed configurations.^74^ See our previous work for the details about the calculation of formation energies and binding energies of CO_2_ and H_2_O molecules on the surface of the model.^12^

### Simulation of the Atomic Configuration

Our model assists in evaluating the probability distribution of the number of nearest neighbor vanadium atoms (*N*_NN_^V^) and S_vac_ sites (*N*_NN_^S_vac_^) around each vanadium atom, named *P*_V–V_(*N*_NN_^V^) and *P*_V–S_vac__(*N*_NN_^S_vac_^), respectively. First, we defined a hexagonal lattice composed of a sandwich structure formed by molybdenum atoms positioned between two layers of sulfur atoms with trigonal-prismatic coordination, where each layer of the constructed lattice consists of 100 × 100 points. In the proposed model, we considered two systems, (i) random and (ii) biased dispersions. (i) For the random dispersion, we randomly replaced molybdenum and sulfur atoms with vanadium atoms and S_vac_ sites, respectively. (ii) For biased dispersion, DFT is used to calculate the formation energies of vanadium and S_vac_ in the MoS_2_ host lattice. For the dispersion of vanadium, four scenarios were considered: V–V pairs are assigned as first neighbors (*d*_V–V_ = 3.21 Å), second neighbors (*d*_V–V_ = 5.52 Å), third neighbors (*d*_V–V_ = 6.35 Å), or beyond third neighbors (*d*_V–V_ > 6.35 Å). The formation energies (*E*_i_^V–V^) for the first three scenarios are calculated to be 0.5062, 0.6566, and 0.6499 eV, respectively. Using a Boltzmann distribution, the acceptance probability (*p*_i_) of each scenario is given by

5 $$p_{i}=\frac{e^{-E_{i}^{\mathrm{VV}}/k_{B}T}}{\sum_{i}e^{-E_{i}^{\mathrm{VV}}/k_{B}T}}$$

where *k*_B_ and *T* are the Boltzmann constant and growth temperature, respectively. If *d*_V–V_ > 6.35 Å, the program accepts the presence of vanadium with an acceptance probability of α_VV_ ranging from 1 to 100%. To introduce S_vac_, we utilized the formation energies of sulfur both with (*E*_i_^S_vac_–V^) and without (*E*_i_^S_vac_–Mo^) binding to vanadium atoms of 2.3700 and 2.6940 eV, respectively. Similarly, we used the Boltzmann distribution to determine the acceptance of S_vac_. Finally, we counted *N*_NN_^V^ and *N*_NN_^S_vac_^ to compute *P*_V–V_(*N*_NN_^V^) and *P*_V–S_vac__(*N*_NN_^S_vac_^). Notably, we used free software Dev-C++ 5.0 (version 4.9.9.2) for programming in C++.

### Photocatalytic CO_2_ Reduction

PC CO_2_ reduction experiments were carried out in a custom-built 7.0 mL stainless steel reactor at room temperature. The reaction duration was 4 h, conducted under the illumination of a commercial 150 W Xe lamp (>320 nm, AM 1.5G) with different intensities from 0.5 to 1.5 sun. The CO signal was recorded by injecting a certain volume (using a Trajan SGE syringe) of the products into an Agilent 6890 GC system equipped with a helium ionization detector and oven operating at 220 °C. Blank tests were also conducted as follows: (i) With photocatalyst/with CO_2_/without light, (ii) With photocatalyst/without CO_2_ (here, we purged instead of flowing CO_2_)/with light, and (iii) Without photocatalyst/with CO_2_/with light.^12,75^ Following this, the values derived from the blank experiments were subtracted from the product yields in the photocatalytic reaction. It should be noted that the contribution of blank tests (i) and (iii) was negligible. To verify the source of the product, an isotope-labeling experiment was conducted by using ^13^CO_2_. Subsequently, the products were analyzed through GC–MS using a Thermo brand instrument (Model: Trace 1300 GC + ISQ MS). AQE was calculated by the following equations

6 $$\mathrm{AQE}=\frac{2\times Y\times N_{A}}{\mathrm{\phi ̅}\times t}$$

which is defined by the ratio of the number of electrons used for CO product yield (*Y*) to the photons incident onto the catalyst (ϕ̅). It should be noted that the numerator contains the number “2” since the CO_2_–to-CO conversion involves only two-electron/proton transfer. *N*_A_ and *t* are Avogadro’s number and irradiation time, respectively. Hence, by considering the overall absorption percentage, *i.e.*,

7 $$IQE=\frac{AQE}{\beta_{\mathrm{AM}\;1.5G}}$$

once can estimate IQE.^7,12^ Further, transferred electron rates per active site are defined as the ratio of the total number of electrons used for CO product yield per second per number of active sites (*N*_S_vac__)­

8 $$R_{\mathrm{CO}}^{S_{\mathrm{vac}}}=\frac{2\times Y\times N_{A}}{S\times t\times N_{S_{\mathrm{vac}}}}$$

where *S* is the exposed area of the film.

### SECM Experiment

The SECM experiments were carried out with a Bruker Dimension Icon, in a PeakForce SECM module equipped with an electrochemical analyzer (CHI760D) and a commercialized nanoelectrode probe (Bruker) operating at −0.4 V versus the Ag pseudoreference electrodes. The solution contained 5 mM [Ru­(NH_3_)_6_]­Cl_3_ and 0.1 M KNO_3_ as a reversible redox mediator and a supporting electrolyte in deionized (DI) water, respectively.^60^ It should be noted that we used analytical approximations for fitting the normalized dimensionless positive feedback currents

9 $$\frac{I^{+}\left(\frac{z}{a}\right)}{I_{0}}=K_{1}^{+}+\frac{K_{2}^{+}}{\left(\frac{z}{a}\right)}+K_{3}^{+}e^{K_{4}^{+}/\left(z/a\right)}$$

where *K*_1_^+^, *K*_2_^+^, *K*_3_^+^, and *K*_4_^+^ are dimensionless constants and $\frac{z}{a}$ , where *a* is the tip radius, is the dimensionless tip–surface distance.^12^ In here, *I*_0_ is the feedback current at $\frac{z}{a}=120$ (*i.e.*, *z* ≫ *a*). Notably, the lateral resolution of our system is about 200 nm.

## Supplementary Material



## Notes

Schematic of the membrane-controlled vapor–liquid–solid growth method; surface topography of the plasma-enhanced atomic layer deposited films; no vanadium signal for conventional vapor–liquid–solid growth by the XPS characterization; thermogravimetric analyses of precursors; effect of the SiO_2_ capping layer thickness on the molybdenum oxidation state; effect of the MoO_3_ layer thickness on the film thickness and vibrational modes; quantitative evidence of wafer-scale uniformity; effect of the growth temperature on the MoS_2_ film; effect of the growth temperature on the vibrational modes of MoS_2_; surface topography of MoS_2_ grown at different temperatures; surface topography of Mo_1–*x*_V_*x*_S_2_ grown with different thicknesses of V_2_O_5_ layer; Bader charge analysis of pristine and doped films; S_vac_ formation energy; overall light absorption; partial density of states; theoretically calculated absorption coefficient; microstructure and stacking order of the pristine MoS_2_ film; microstructure and stacking order of the Mo_0.7_V_0.3_S_2_ film; growth mechanism; element loss during the ultraconfined membrane-controlled vapor–liquid–solid growth; impact of NaF thickness; grown film by using NaCl; gas chromatography signal of the CO product; ultraviolet photoelectron spectroscopy measurement; absorption edge; table of fitted values from Raman spectra; and supporting references (PDF)
