# Supplementary material for: Capped Vapor–Liquid–Solid Growth of Vanadium-Substituted Molybdenum Disulfide Ultrathin Films for Enhanced Photocatalytic Activity
Source: ACS Nano. 2026 Jan 7;20(2):2211–24. doi: 10.1021/acsnano.5c17367 (PMC12825380; doi:10.1021/acsnano.5c17367)
Supplement: Supplementary file 1 [file nn5c17367_si_001.pdf]

Supporting Information

Capped Vapor–Liquid–Solid Growth of Vanadium-Substituted Molybdenum Disulfide Ultrathin Films for Enhanced Photocatalytic Activity

Pin-Pin Huang,[†,‡,δ] Mohammad Qorbani,*[§,||,¶,#,δ] Ying-Ti Hung,[¶,#,⊥,δ] Ying-Ren Lai,[¶,#,δ] Amr Sabbah,[¶,#,@,∇] Mao-Feng Tseng,[Δ] Chih-Yang Huang,[¶,#,◊,○] Sumangaladevi Koodathil,[¶,#,◊,○] Septia Kholimatussadiah,[¶,□,♠,♣] Mahmoud Kamal Hussien,[¶,#,∇,♦] Tzu-Hsuan Feng,[¶] Yo-Hsun Liu,[¶,♪] Hsin Wang,[†,⊥] Jia-Wei Lin,[¶,#] Chen-Hao Wang,[♪] Chih-I Wu,[♥] Michitoshi Hayashi,*[¶,#,⊤] Kuei-Hsien Chen,*[†,¶] and Li-Chyong Chen*[¶,#,□]

[†]Institute of Atomic and Molecular Sciences, Academia Sinica, Taipei 10617, Taiwan
[‡]Department of Chemistry, National Taiwan Normal University, Taipei 10610, Taiwan
[§]Undergraduate Program of Electro-Optical Engineering, National Taiwan Normal University, Taipei 11677, Taiwan
[||]Institute of Electro-Optical Engineering, National Taiwan Normal University, Taipei 11677, Taiwan
[¶]Center for Condensed Matter Sciences, National Taiwan University, Taipei 10617, Taiwan
[#]Center of Atomic Initiative for New Materials, National Taiwan University, Taipei 10617, Taiwan
[⊥]Department of Materials Science and Engineering, National Taiwan University, Taipei 10617, Taiwan
[@]Tabbin Institute for Metallurgical Studies, Tabbin, Helwan 109, Cairo 11421, Egypt
[∇]Interdisciplinary Research Center for Hydrogen Technologies and Carbon Management (IRC-HTCM), King Fahd University of Petroleum & Minerals, Dhahran, 31261 Saudi Arabia
[Δ]School of Electrical, Computer and Energy Engineering, Arizona State University, Tempe, AZ, 85287–5706, USA
[◊]Molecular Science and Technology Program, Taiwan International Graduate Program, Academia Sinica, Taipei 11529, Taiwan
[○]International Graduate Program of Molecular Science and Technology, National Taiwan University, Taipei, 10617, Taiwan
[□]Department of Physics, National Taiwan University, Taipei 10617, Taiwan
[♠]Nano Science and Technology, Taiwan International Graduate Program, Academia Sinica, Taipei 11529, Taiwan
[♣]Institute of Physics, Academia Sinica, Taipei 11529, Taiwan
[♦]Department of Chemistry, Faculty of Science, Assiut University, Assiut 71516, Egypt
[♪]Department of Materials Science and Engineering, National Taiwan University of Science and Technology, Taipei 106335, Taiwan
[♥]Graduate Institute of Photonics and Optoelectronics, National Taiwan University, Taipei 10617, Taiwan
[⊤]National Center for Theoretical Sciences, Taipei 10617, Taiwan
[δ]These authors contributed equally

Corresponding E-mail addresses: qorbani@ntnu.edu.tw (M. Qorbani); atmyh@ntu.edu.tw (M. Hayashi); chenkh@pub.iams.sinica.edu.tw (K.-H. Chen); chenlc@ntu.edu.tw (L.-C. Chen)

Content

Supporting Figures....3

Figure S1. Schematic of the membrane-controlled vapor–liquid–solid growth method....3

Figure S2. Surface topography of the plasma-enhanced atomic layer deposited films....3

Figure S3. No vanadium signal for conventional vapor–liquid–solid growth. 4

Figure S4. Thermogravimetric analyses....5

Figure S5. Effect of the SiO_2 capping layer thickness on the molybdenum oxidation state....6

Figure S6. Effect of the MoO_3 layer thickness on the film thickness and vibrational modes....7

Figure S7. Effect of the growth temperature on the MoS_2 film. 8

Figure S8. Quantitative evidence of wafer-scale uniformity. 9

Figure S9. Effect of the growth temperature on the vibrational modes of MoS_2. 10

Figure S10. Surface topography of MoS_2 grown at different temperatures....11

Figure S11. Impact of V_2O_5 thickness....11

Figure S12. Surface topography of $Mo_{1-x}V_xS_2$ grown with different thicknesses of V_2O_5 layer. 12

Figure S13. Bader charge analysis....12

Figure S14. S_{vac} formation energy. 13

Figure S15 Overall light absorption....14

Figure S16 Partial density of states....15

Figure S17 Theoretically calculated absorption coefficient. 15

Figure S18 Microstructure and stacking order of the pristine MoS_2 film. 16

Figure S19 Microstructure and stacking order of the $Mo_{0.7}V_{0.3}S_2$ film. 17

Figure S20 Growth mechanism. 18

Figure S21 Element loss during the ultraconfined membrane-controlled vapour–liquid–solid growth. 19

Figure S22 The impact of NaF thickness. 19

Figure S23 Grown film by using NaCl....20

Figure S24 Gas chromatography signal of the CO product....20

Figure S25 Ultraviolet photoelectron spectroscopy measurement....21

Figure S26 Absorption edge. 22

Supporting Tables....23

Table S1 Raman data. 23

Supporting References....24

Supporting Figures

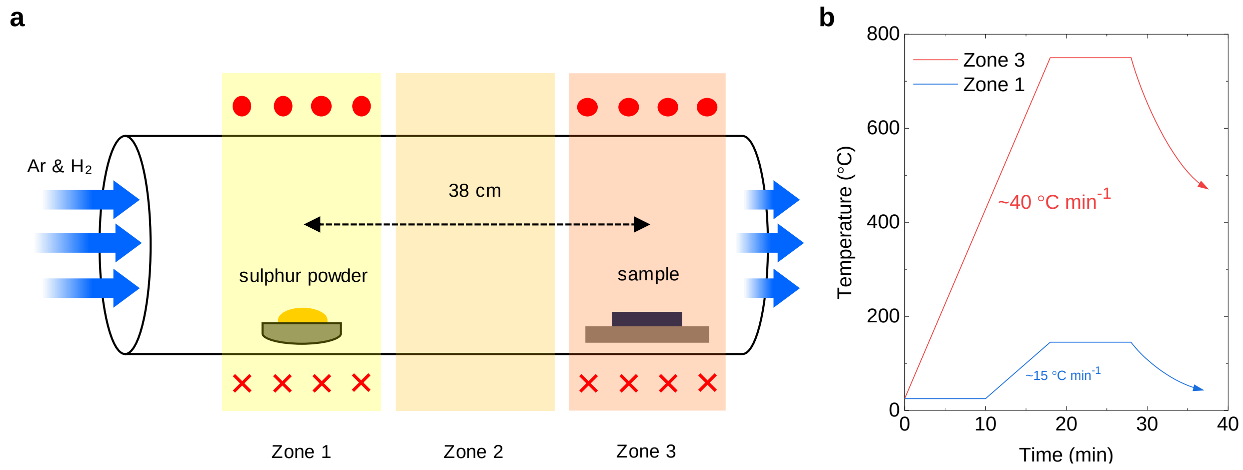

Figure S1. Schematic of the membrane-controlled vapor–liquid–solid growth method. (**a**) The setup of the growth method. 300 mg sulphur powder and sample (solid precursors on the SiO_2/Si substrate) were put in an alumina boat and on a quartz plate in the centers of zone 1 and zone 3, respectively. (**b**) Temperature ramping profiles of zone 1 (blue line) and zone 3 (red line). It is worth noting that the final step involves a natural cooling process.

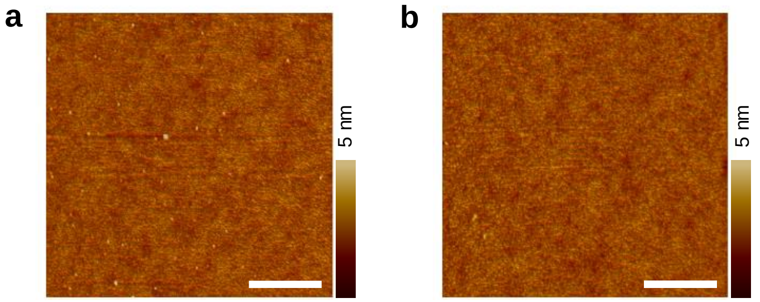

Figure S2. Surface topography of the plasma-enhanced atomic layer deposited films. (**a–b**) Atomic force microscopy (AFM) images of the MoO_3 and V_2O_5 (on MoO_3) layers grown by the plasma-enhanced atomic layer deposition (PEALD), respectively. Scale bar = 1 µm. Notably, we optimized the growth parameters of the PEALD to have a smooth surface.

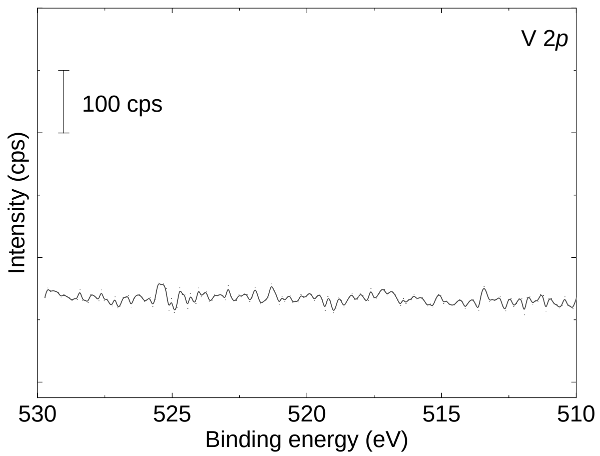

Figure S3. No vanadium signal for conventional vapor–liquid–solid growth. X-ray photoelectron spectroscopy (XPS) spectrum of V 2*p* from the grown film without the SiO_2 capping layer.

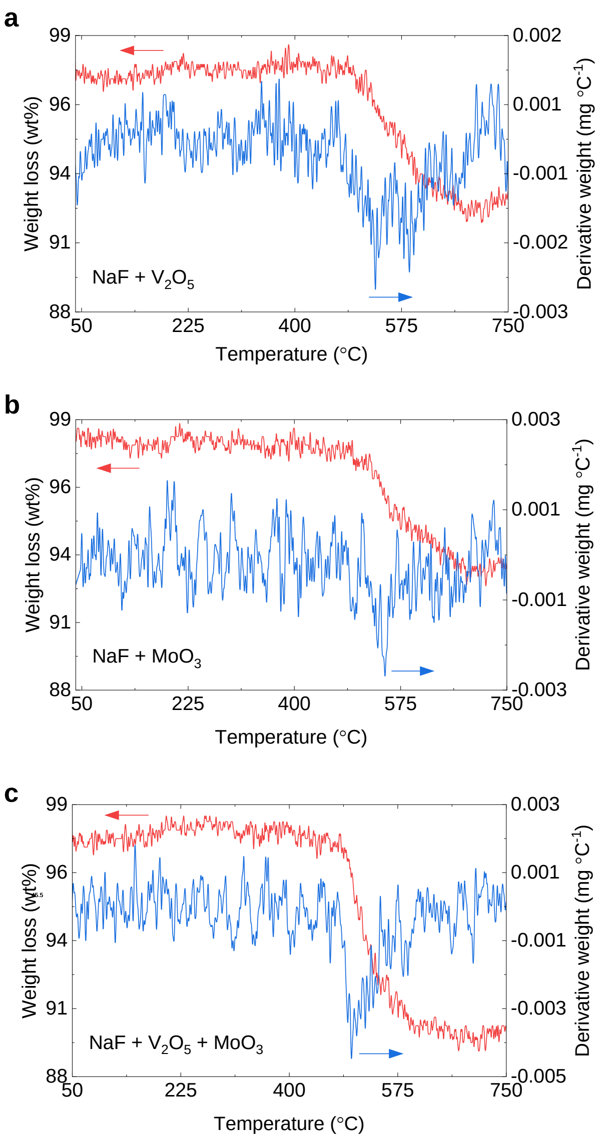

Figure S4. Thermogravimetric analyses. (**a**–**c**) Mass loss and derivative weight of NaF + V_2O_5, NaF + MoO_3, and NaF + V_2O_5 + MoO_3, respectively. Thermogravimetric analysis results show the onset of decomposition temperatures at ~490 (in NaF + V_2O_5 curve) and ~510 ºC (in NaF + MoO_3 curve) could be assigned to the formation of gas phase VF_5 and MoO_2F_2 compounds, respectively.[1] The NaF + V_2O_5 + MoO_3 curve also displays a sharp exothermal peak with a larger mass loss compared with NaF + V_2O_5 and NaF + MoO_3 curves. It implies the simultaneous pumping out of VF_5 and MoO_2F_2 compounds from the system.

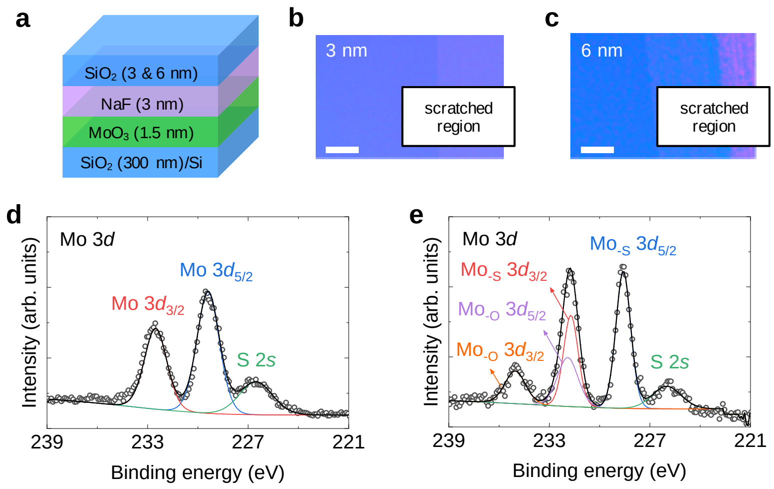

Figure S5. Effect of the SiO_2 capping layer thickness on the molybdenum oxidation state. (**a**) Schematic structure of the solid precursors used for the study of the SiO_2 layer thickness, (**b–c**) Optical microscopy (OM) images, and (**d–e**) XPS spectra of the MoS_2 layers grown by 3-nm and 6-nm SiO_2 capping layers after growth process at 750 °C for 10 minutes, respectively. Scale bar = 20 μm.

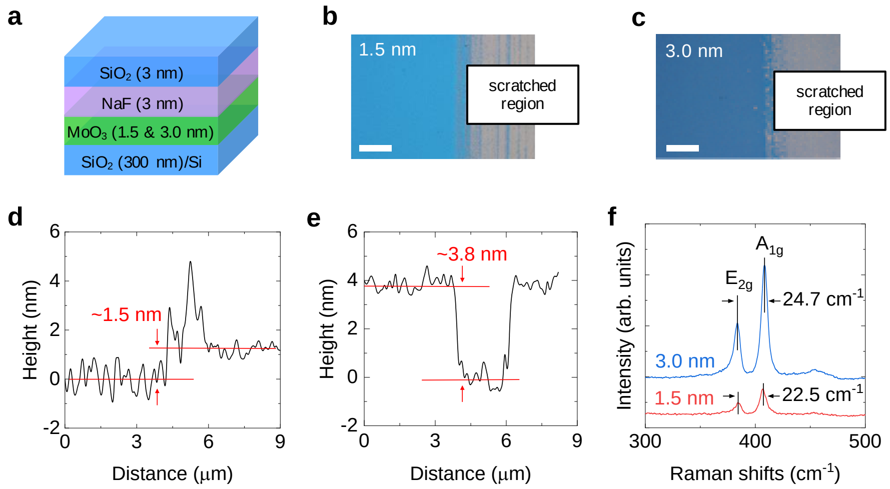

Figure S6. Effect of the MoO_3 layer thickness on the film thickness and vibrational modes. (**a**) Schematic structure of the solid precursors used for the study of the MoO_3 layer thickness, (**b–c**) optical microscopy images, (**d–e**) AFM height profiles, and (**f**) Raman spectra of the MoS_2 layers grown by 1.5 and 3.0 nm MoO_3 layers after growth process at 750 °C for 10 minutes, respectively. Scale bar = 20 μm.

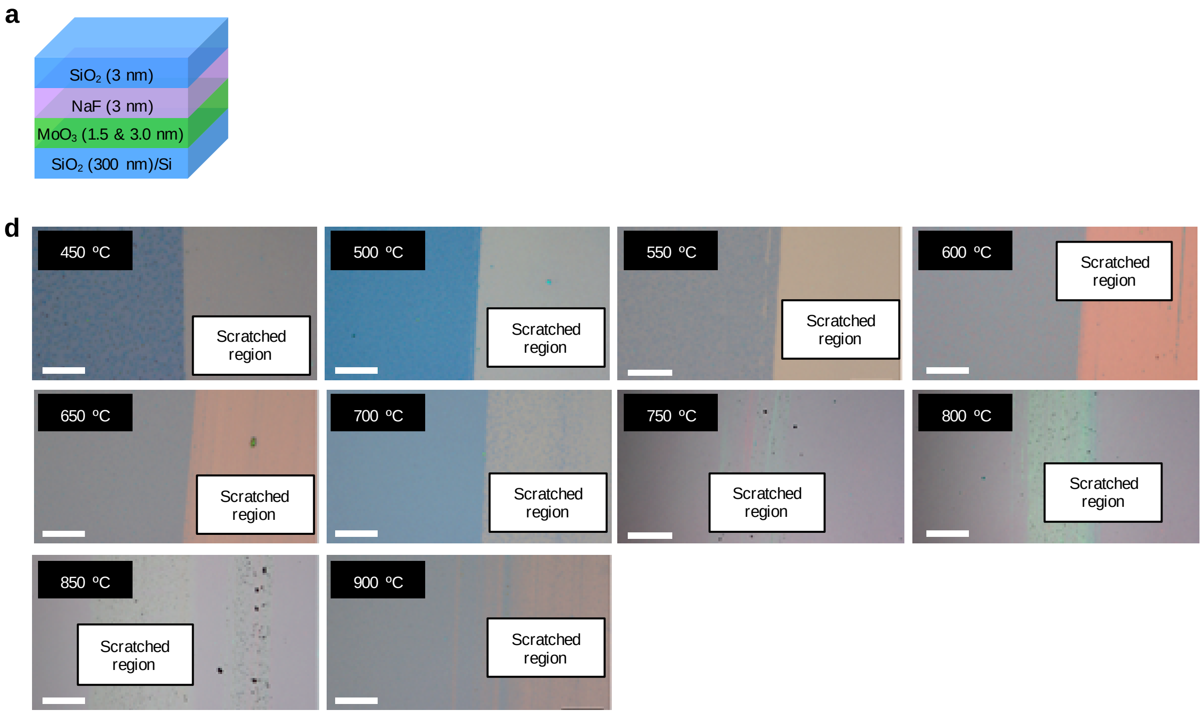

Figure S7. Effect of the growth temperature on the MoS_2 film. (**a**) Schematic structure of the solid precursors used for the study of the effect of the growth temperature, (**b**) optical microscopy images of the grown films at different growth temperatures. Scale bar = 20 μm. We observed that the grown films at low growth temperatures, *i.e.*, < 750 °C, are hard and it is difficult to scratch implying a partial sulfurization due to the low rate of S diffusion into the SiO_2 capping layer. On the other hand, we observed the presence of non-continuous film that can be assigned to the upward diffusion of eutectic liquid over the SiO_2 capping layer at high growth temperatures, *i.e.*, > 750 °C. Therefore, the growth temperature of 750 °C is found as the optimized temperature to grow a uniform and high-quality film.

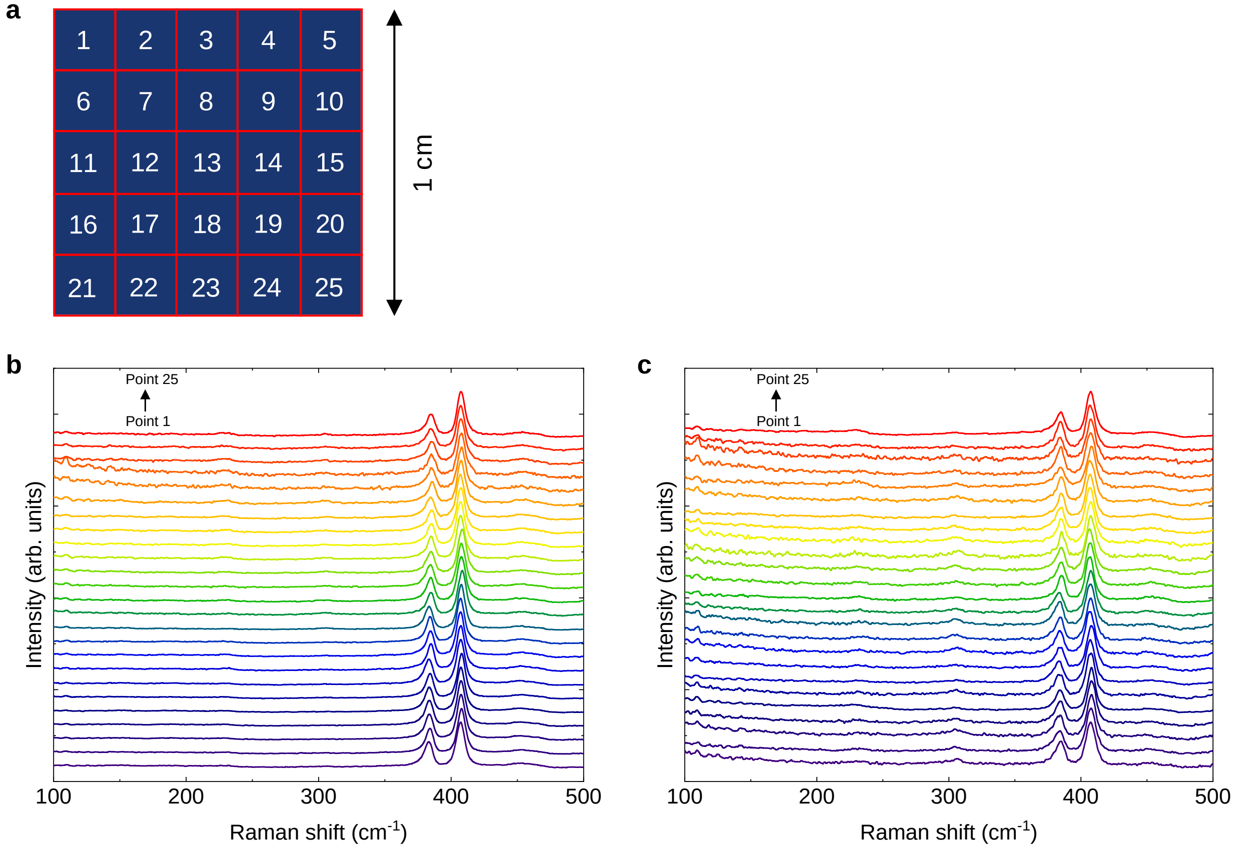

Figure S8. Quantitative evidence of wafer-scale uniformity. (**a**) Schematic positions of Raman data acquisition. (**b**–**c**) Raman spectra from 25 points of MoS_2 and $Mo_{1-x}V_xS_2$ films, respectively.

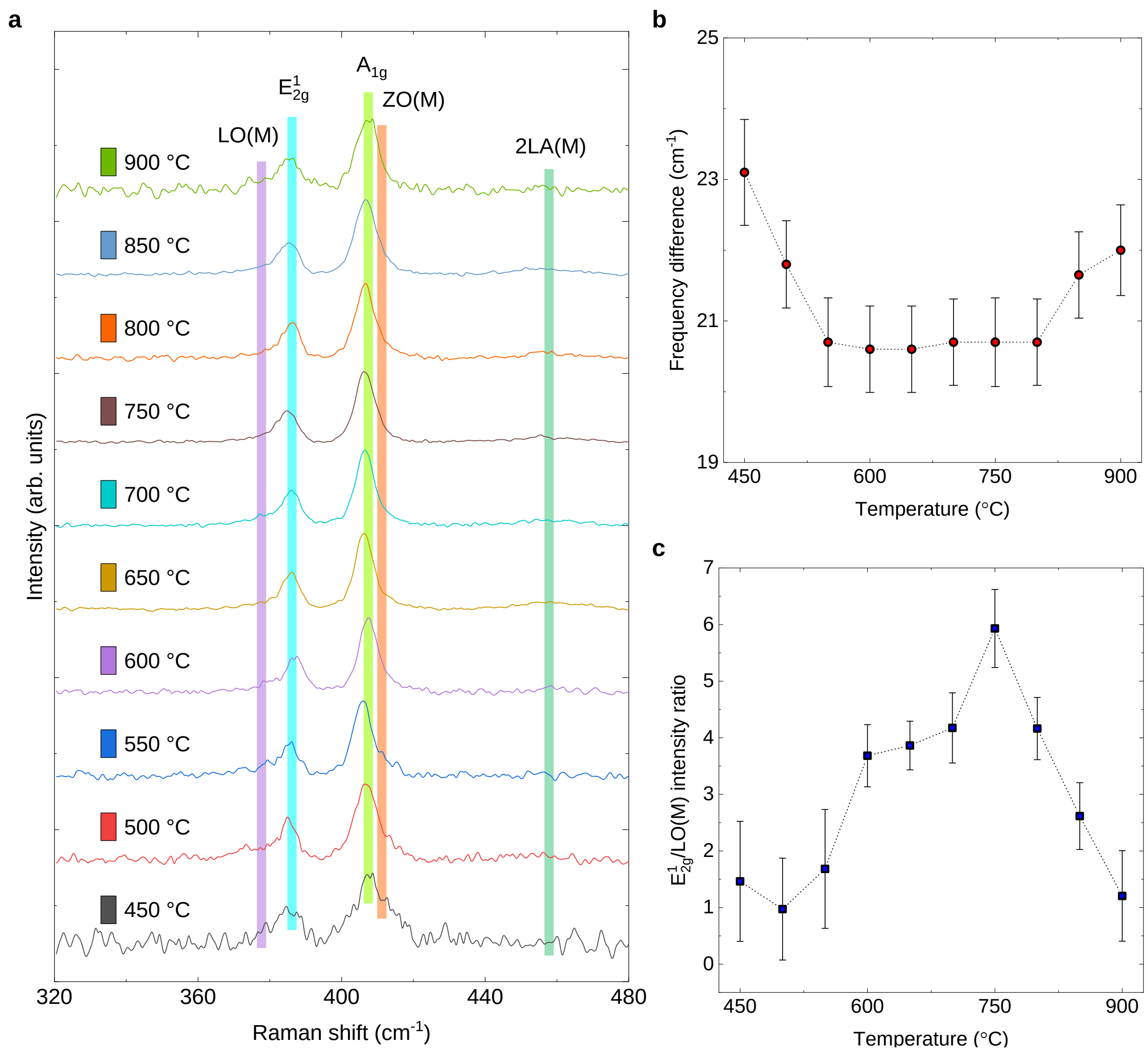

Figure S9. Effect of the growth temperature on the vibrational modes of MoS_2. (**a**) Raman spectra containing $LO(M)$, E^1_{2g}, A_{1g}, $ZO(M)$, and $2LA(M)$ peaks stand for the longitudinal optical, in-plane, out-of-plane, out-of-plane optical, and longitudinal acoustic modes, respectively. (**b–c**) Frequency difference between A_{1g} and E^1_{2g} modes, *i.e.*, $\omega(A_{1g}) - \omega(E^1_{2g})$, and $E^1_{2g}/LO(M)$ ratio as a function of growth temperature. It should be noted that we used a Lorentzian function to fit the Raman spectra.

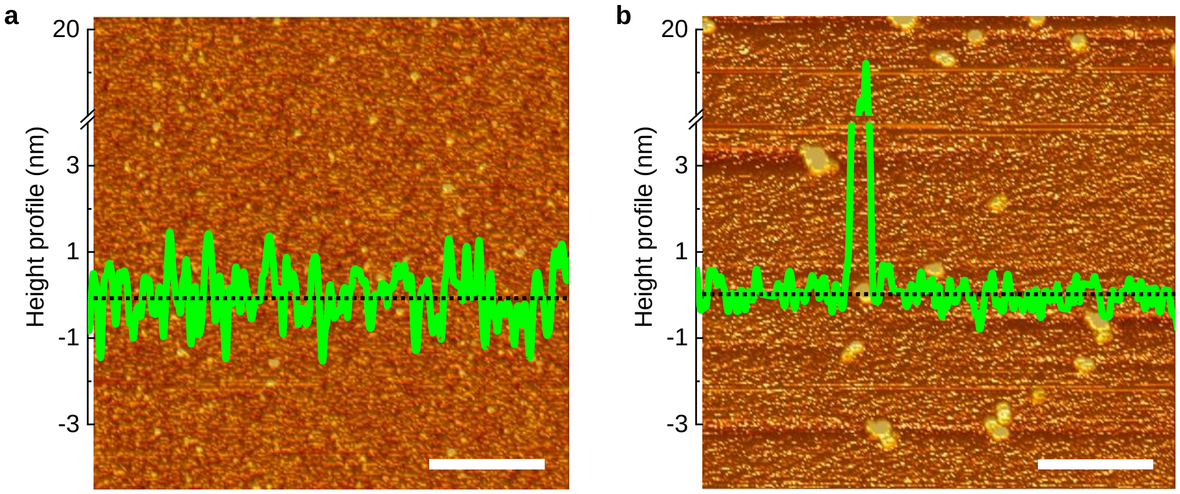

Figure S10. Surface topography of MoS_2 grown at different temperatures. (**a**–**b**) AFM images and height profiles of the samples grown at temperatures of 750 ºC and 800 ºC, respectively. Scale bar = 1 μm.

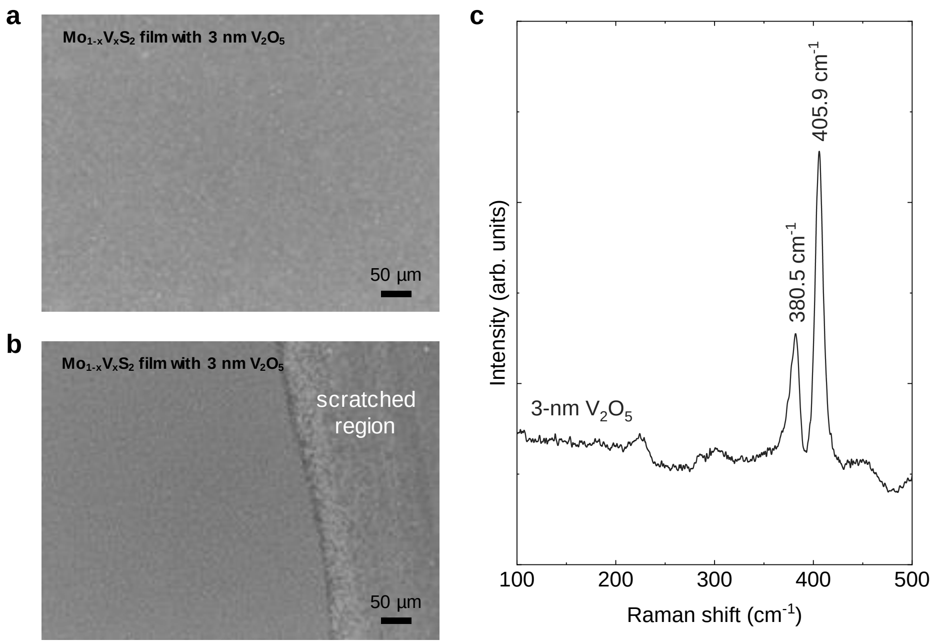

Figure S11. Impact of V_2O_5 thickness. (**a**–**b**) Grayscale optical microscopy images of two different $Mo_{1-x}V_xS_2$ films grown by using a 3-nm V_2O_5 solid layer. (**c**) Raman spectrum of the $Mo_{1-x}V_xS_2$ film grown by using a 3-nm V_2O_5 solid layer. This thicker layer was found to produce a rough surface. However, it's worth noting that the Raman peaks associated with this sample were consistent with those observed in other vanadium-incorporated films.

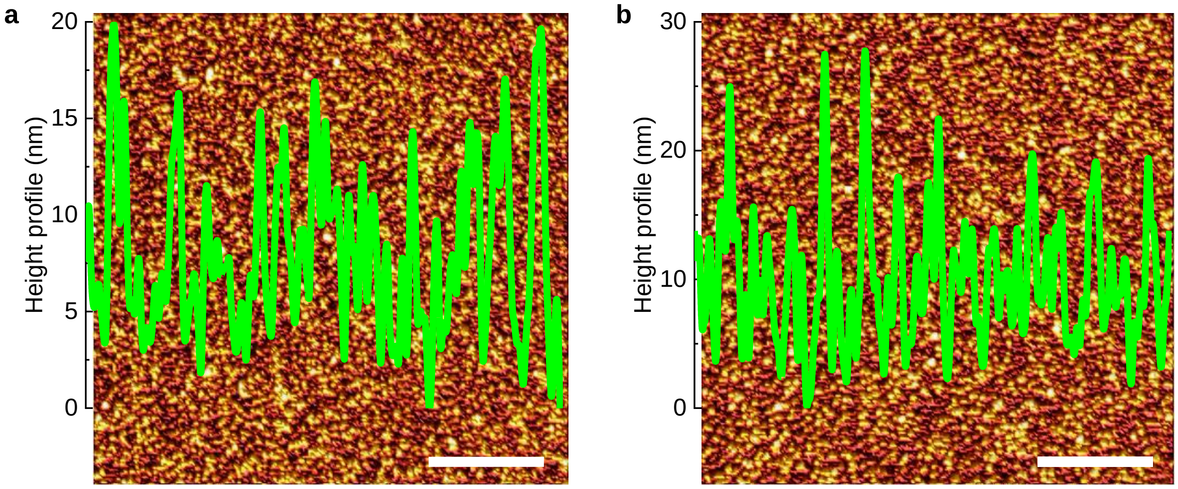

Figure S12. Surface topography of $Mo_{1-x}V_xS_2$ grown with different thicknesses of V_2O_5 layer. (**a**–**b**) AFM images and height profiles of the samples grown at V_2O_5 layer thicknesses of 4 and 6 nm, respectively. Scale bar = 1 μm.

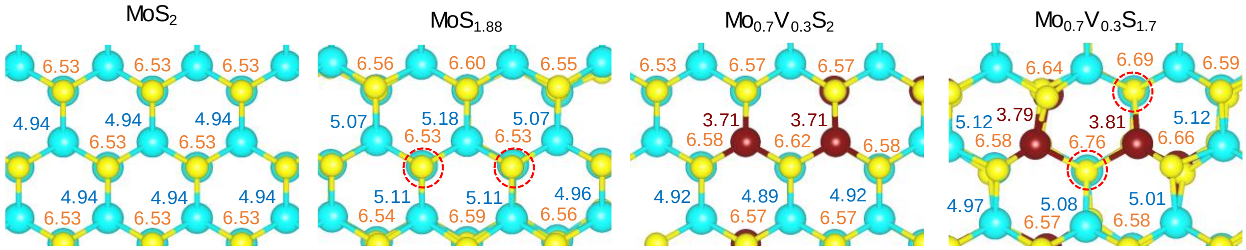

Figure S13. Bader charge analysis. Surface Bader charges distributions of the 2L $Mo_{1-x}V_xS_2$. Blue-, yellow-, and wine-filled circles stand for Mo, S, and V atoms, respectively.

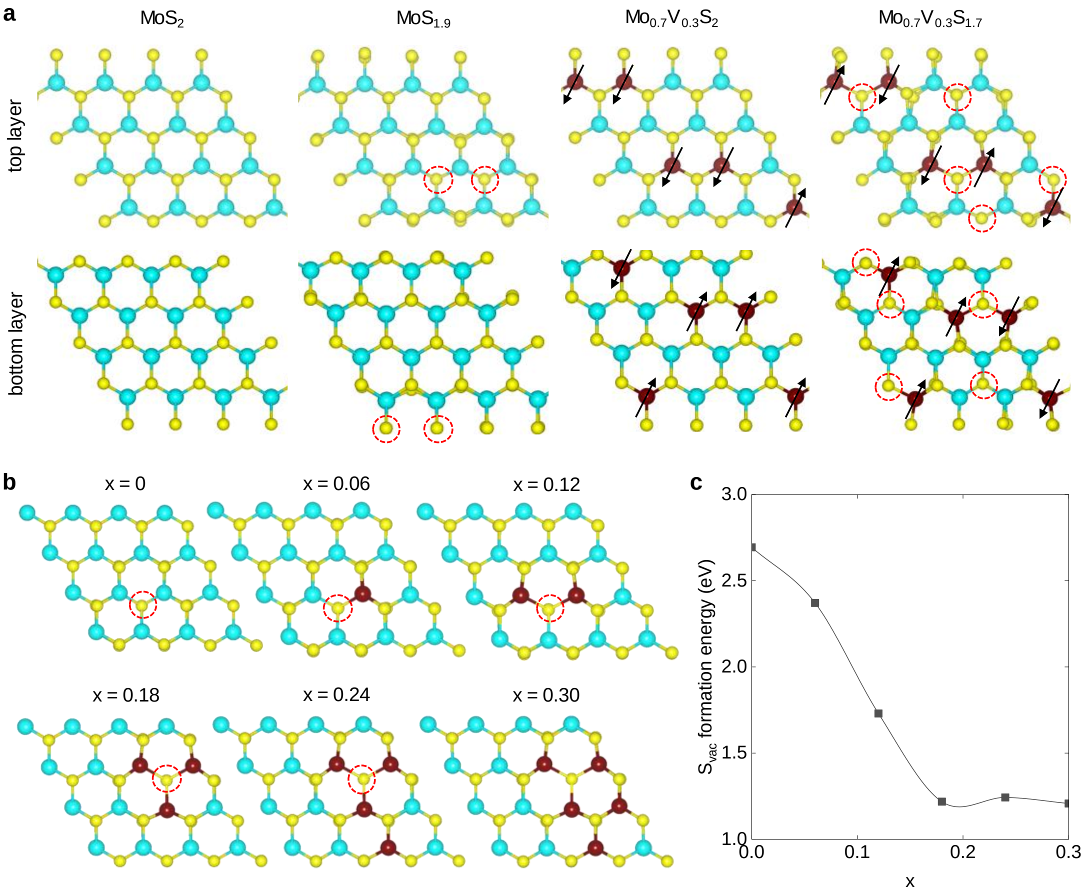

Figure S14. S_{vac} formation energy. (**a**) Density functional theory (DFT) relaxed configurations of bilayer S_{vac}-$\mathbf{Mo_{1\text{-}x}V_xS_2}$. The formation energy of S_{vac} is 2.67 to 1.20 eV for x = 0 and 0.3, respectively. The spins of vanadium atoms and S_{vac} are shown by arrows and dashed circles, respectively. In the $Mo_{1\text{-}x}V_xS_2$ system, the spin degree of freedom plays a crucial role due to the deficiency of one valence electron in a vanadium atom compared to molybdenum atoms.[2-7] Indeed, this is significant for its potential application as a 2D dilute magnetic semiconductor.[8, 9] Our findings demonstrate that both 2L MoS_2 and S_{vac}-MoS_2 display paired electrons, indicative of diamagnetic characteristics. On the contrary, in the instances of 2L $Mo_{1\text{-}x}V_xS_2$, each vanadium atom harbors an unpaired electron, leading to an overall antiferromagnetic property attributed to interlayer interactions.[2] Upon introducing S_{vac} into the 2L $Mo_{1\text{-}x}V_xS_2$ (i.e., S_{vac}-$Mo_{1\text{-}x}V_xS_2$), the spin directions undergo a transformation from parallel to antiparallel when an S_{vac} is positioned between two adjacent vanadium atoms. (**b**–**c**) DFT relaxed configurations and formation energy of 1L S_{vac}-$Mo_{1\text{-}x}V_xS_2$, respectively. S_{vac} is shown by dashed circles. The line in (**c**) is added as a guide to the eye.

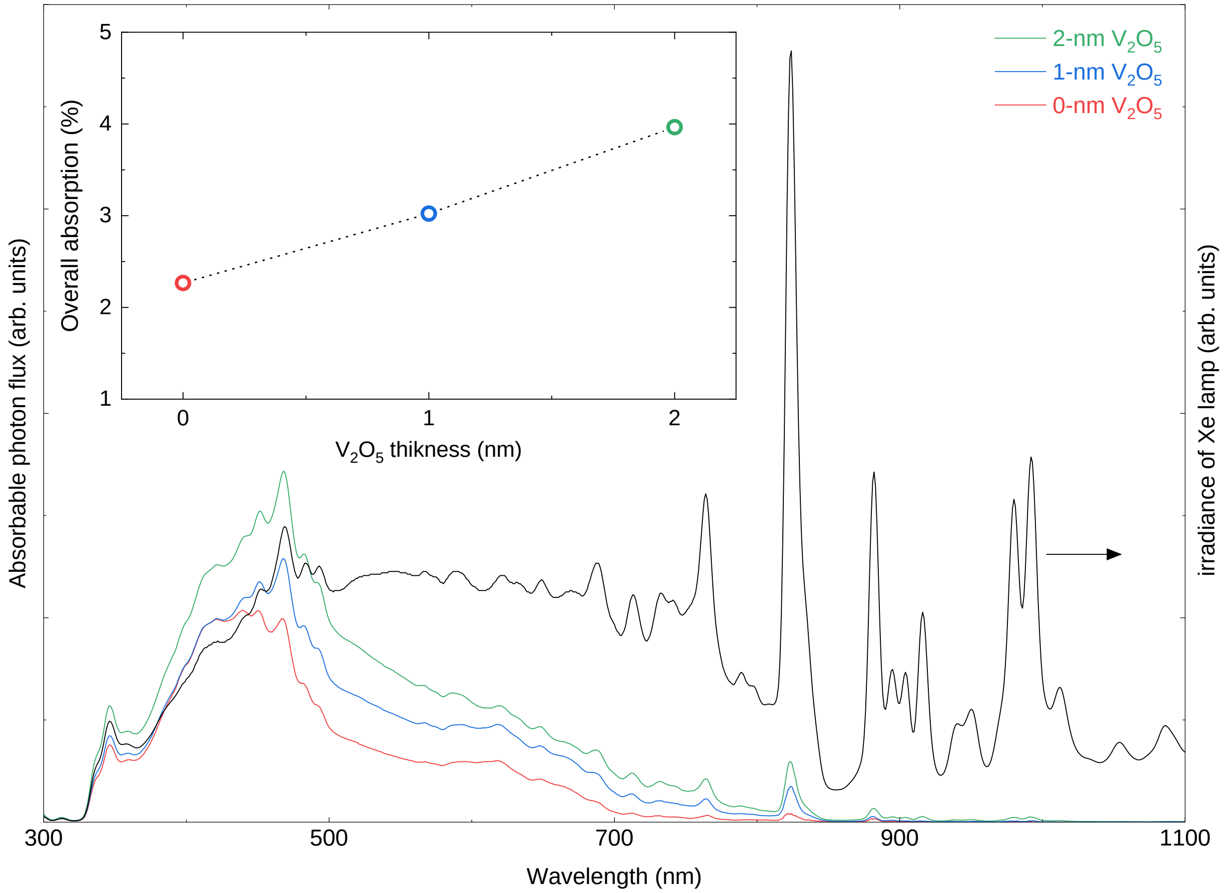

Figure S15 Overall light absorption. Absorbable photon flux of the films without V_2O_5 (pristine MoS_2) and with 1 and 2 nm V_2O_5 layer ($Mo_{1-x}V_xS_2$) and the irradiance of the Xe lamp (black line) spectrum. Inset shows the corresponding overall absorption.[10]

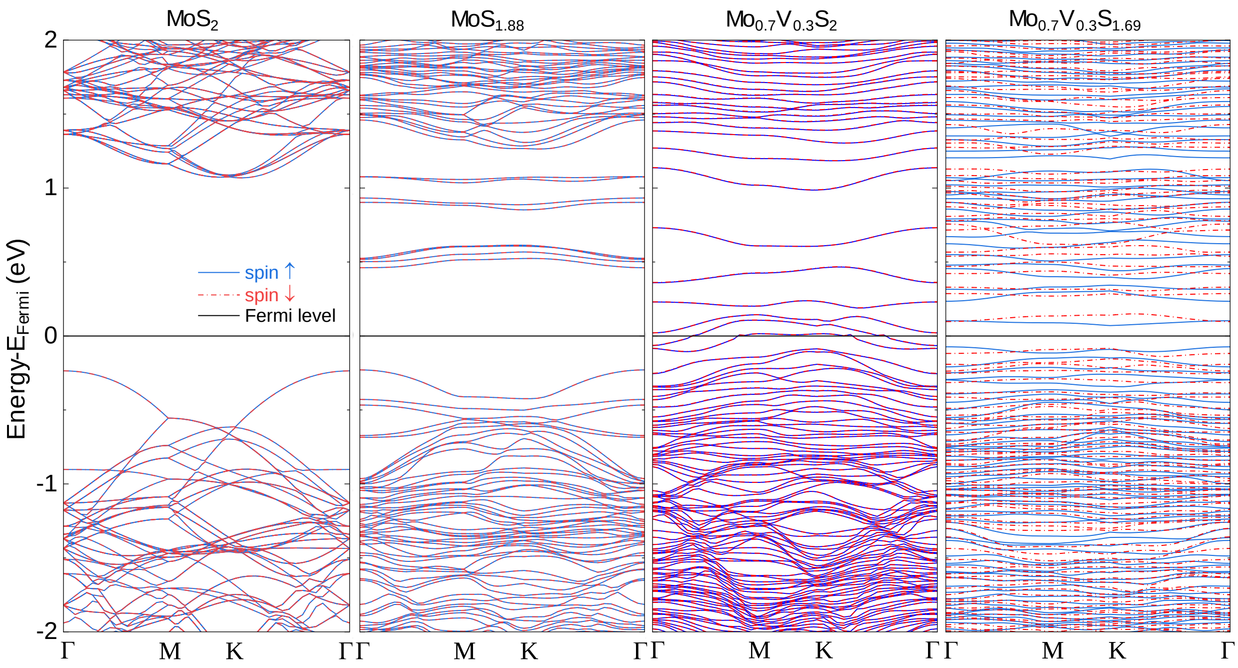

Figure S16 Partial density of states. DFT-calculated partial density of states (PDOS) of spin-up and spin-down levels for bilayer pristine MoS_2, S_{vac}-MoS_2 ($MoS_{1.9}$), $Mo_{1-x}V_xS_2$ ($Mo_{0.7}V_{0.3}S_2$), and S_{vac}-$Mo_{1-x}V_xS_2$ ($Mo_{0.7}V_{0.3}S_{1.7}$) films along the high-symmetry points (*Γ*–*M*–*K*–*Γ*).

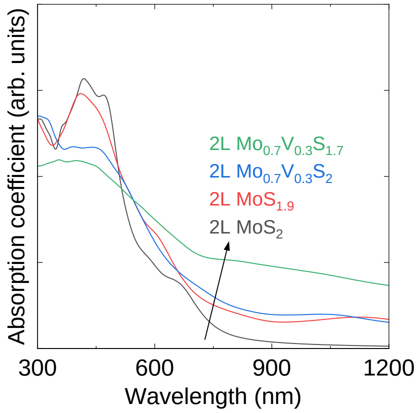

Figure S17 Theoretically calculated absorption coefficient. DFT-calculated absorption coefficient for bilayer pristine MoS_2, S_{vac}-MoS_2 ($MoS_{1.9}$), $Mo_{1-x}V_xS_2$ ($Mo_{0.7}V_{0.3}S_2$), and S_{vac}-$Mo_{1-x}V_xS_2$ ($Mo_{0.7}V_{0.3}S_{1.7}$) films.

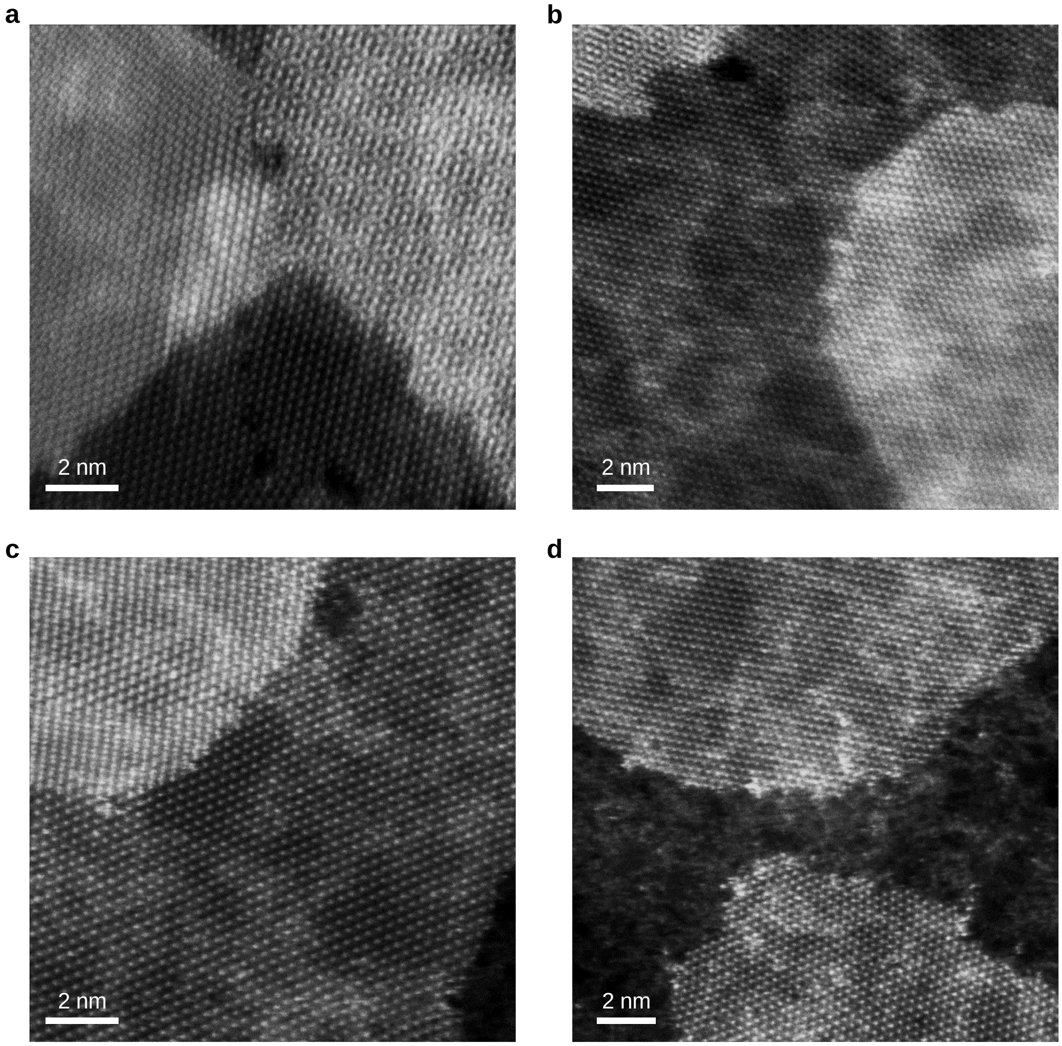

Figure S18 Microstructure and stacking order of the pristine MoS_2 film. (**a–d**) ADF-STEM images from different regions. Notably, the ADF-STEM images were Gaussian blurred with the sigma value of two.

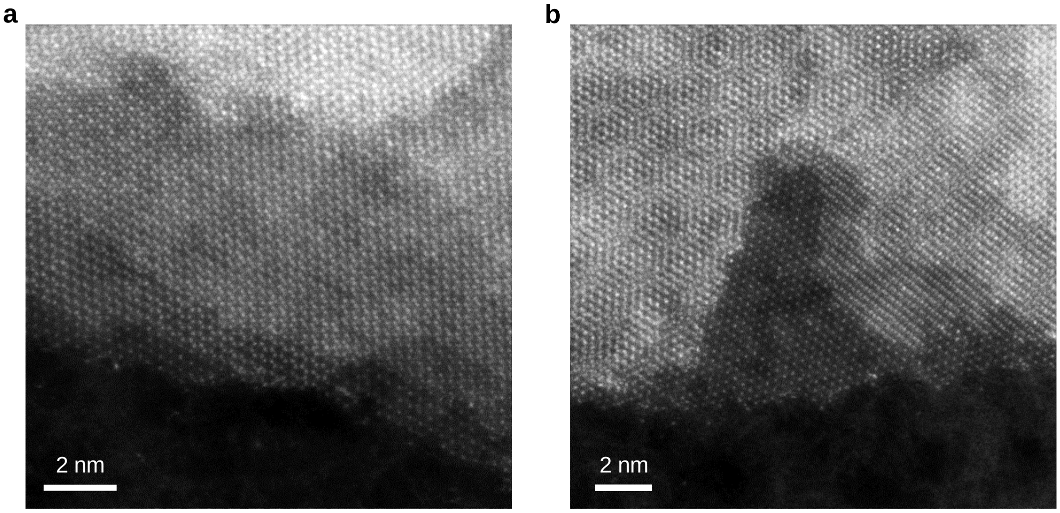

Figure S19 Microstructure and stacking order of the $Mo_{0.7}V_{0.3}S_2$ film. (**a**–**b**) ADF-STEM images from different regions near the grain boundaries. Notably, the ADF-STEM images were Gaussian blurred with the sigma value of two.

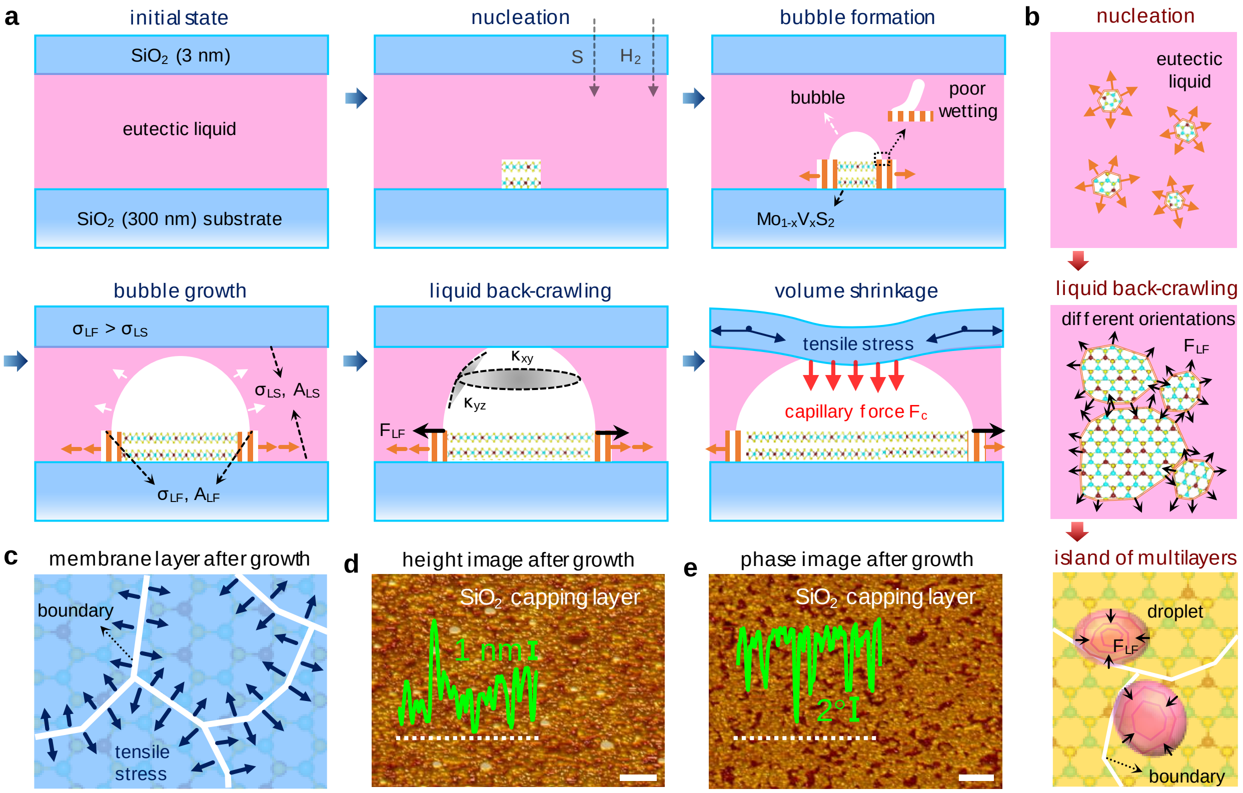

Figure S20 Growth mechanism. (**a**–**b**) Cross-sectional and top-view growth schematic, respectively. (**c**) Schematic of the SiO_2 membrane layer morphology after the growth process. (**d**–**e**) AFM height and phase images of the SiO_2 membrane layer after the growth process, respectively. Scale bar = 100 nm. Orange arrows, lines, and vertical pattern-filled rectangles show the growth direction. Black arrows show the driving force F_{LF} of the liquid motion. K_{yz} and K_{xy} are the curvatures in the vertical and horizontal directions, respectively. σ_{LF} (A_{LF}) and σ_{LS} (A_{LS}) are the interfacial free energies (areas) of the liquid–film and liquid–SiO_2 interfaces, respectively. Dark blue and red arrows show the direction of tensile stress and capillary force, respectively. Blue-, yellow-, and wine-filled circles stand for Mo, S, and V atoms.

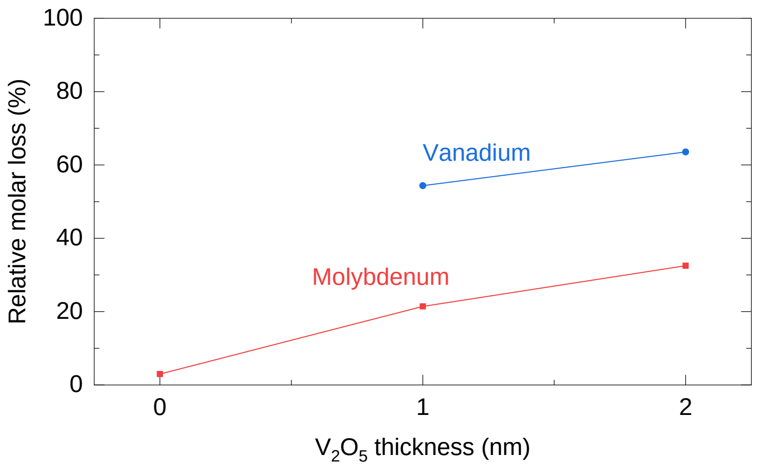

Figure S21 Element loss during the ultraconfined membrane-controlled vapour–liquid–solid growth. Relative molar loss of molybdenum and vanadium as a function of V_2O_5 thickness. The relative molar loss is calculated based on the molar density and thickness of the precursors and grown 2L film. It shows that more vanadium results in the molybdenum loss that would be assigned to the formation of low-bowling point molybdenum vanadium fluoride compound.

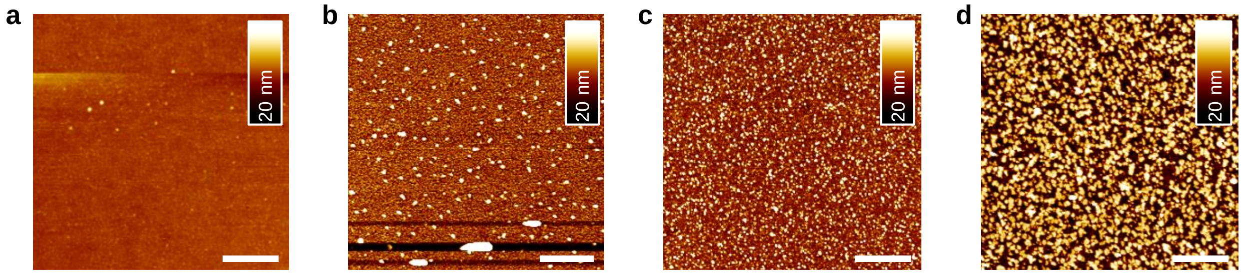

Figure S22 The impact of NaF thickness. (a–d) AFM images of the grown films by using 2, 3, 7, and 11 nm thick NaF solid layer, respectively. Scale bar = 1 µm.

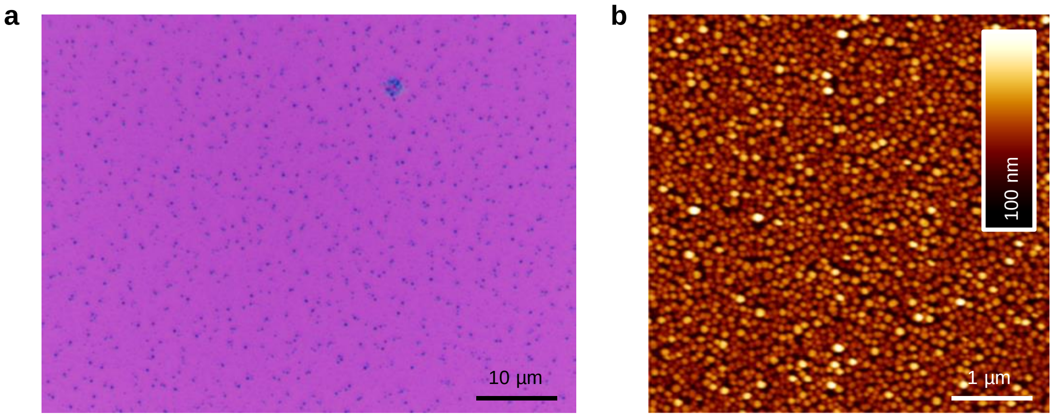

Figure S23 Grown film by using NaCl. (a–b) Optical microscopy and AFM images of the grown films by using 5-nm thick NaCl solid layer.

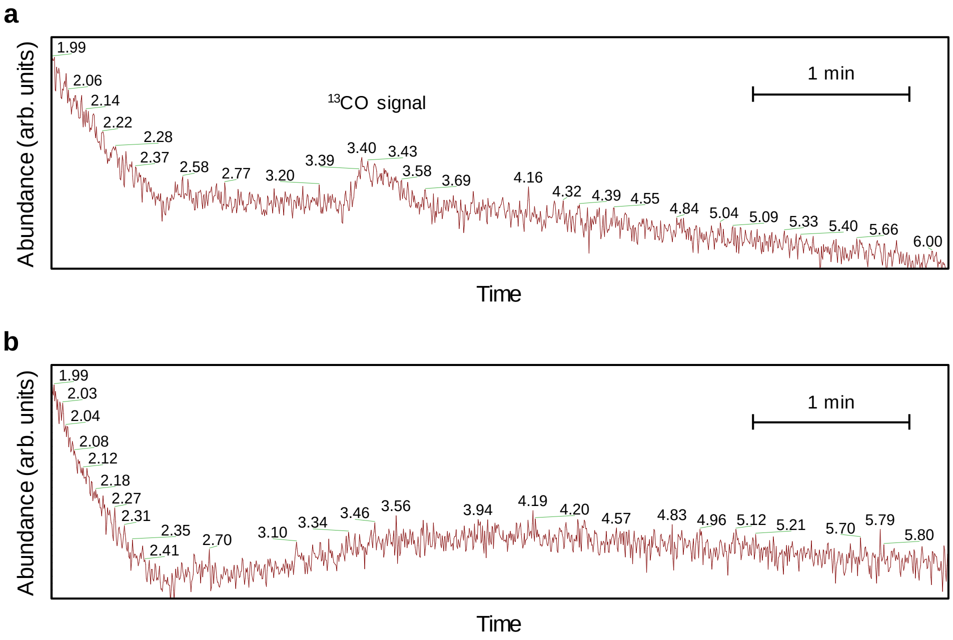

Figure S24 Gas chromatography signal of the CO product. (a–b) The gas chromatograph (GC) signal of the isotope tracer $^{13}CO_2$ and background measurements, respectively.

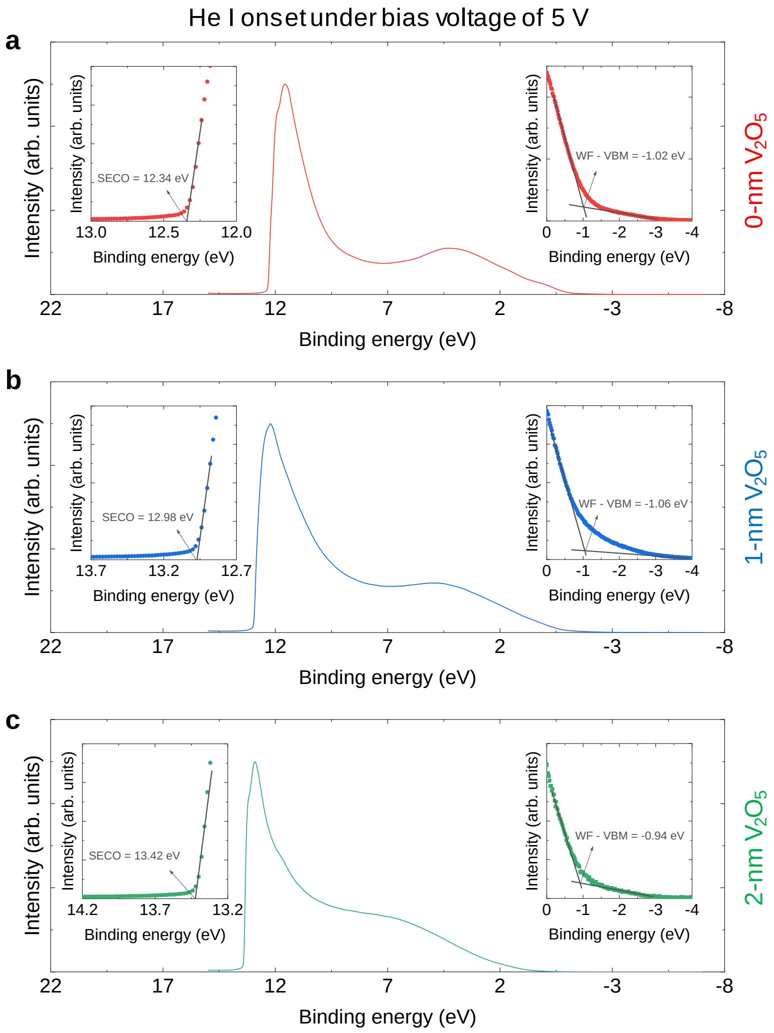

Figure S25 Ultraviolet photoelectron spectroscopy measurement. (a–c) He I ($h\nu$ = 21.2 eV) ultraviolet photoelectron spectroscopy (UPS) measurements to find the secondary electron cut-off (SECO), work function (WF), and valence band maximum (VBM) of the films without V_2O_5 (pristine MoS_2) and with 1 and 2 nm V_2O_5 layer ($Mo_{1-x}V_xS_2$), respectively.

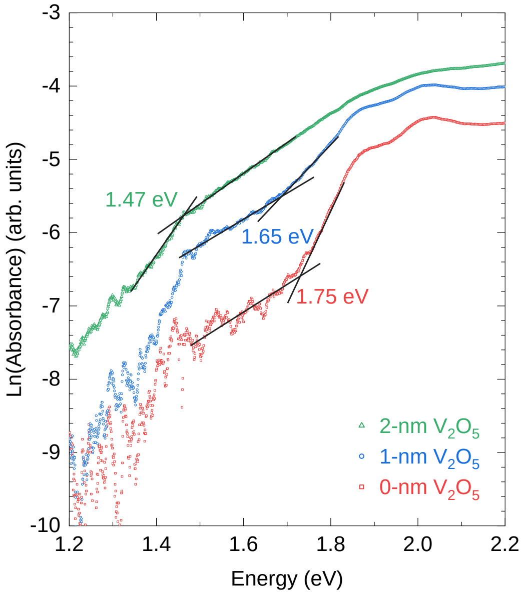

Figure S26 Absorption edge. Logarithm of absorbance versus energy for the films without V_2O_5 (pristine MoS_2) and with 1 and 2 nm V_2O_5 layer ($Mo_{1-x}V_xS_2$), respectively.

Supporting Tables

Table S1 Raman data. FWHM and frequency difference $\omega(A_{1g}) - \omega(E_{2g}^1)$ for pristine MoS_2 and $Mo_{1-x}V_xS_2$ films are extracted by fitting the Raman spectra shown in **Figure 2d** (main Manuscript) with a Lorentzian function. It should be noted that the frequency difference is 21.6 cm^{-1} for the pristine MoS_2, which is consistent with a 2L MoS_2.[11] Moreover, $Mo_{1-x}V_xS_2$ films show a larger frequency difference of 24.9 cm^{-1}. Although the $Mo_{1-x}V_xS_2$ film thickness is almost the same as that of the pristine one, it cannot be assigned to the enhanced number of layers since V incorporation provides additional $E_{2g}^1(\Gamma)_{V-S}$ peaks. It implies the in-plane $E_{2g}^1(\Gamma)_{Mo/V-S}$ peak is due to the overlap of Mo–S and V–S vibrational modes.

**Sample**	**FWHM of E_{2g}^1 (cm^{-1})**	**FWHM of A_{1g} (cm^{-1})**	**$\omega(A_{1g}) - \omega(E_{2g}^1)$ (cm^{-1})**
0-nm V_2O_5	6.7 ± 0.4	6.6 ± 0.2	21.5
1-nm V_2O_5	13.1 ± 0.2	8.7 ± 0.2	24.5
2-nm V_2O_5	14.6 ± 0.5	9.4 ± 0.2	24.9

Supporting References

1. Chang, M. C.; Ho, P. H.; Tseng, M. F.; Lin, F. Y.; Hou, C. H.; Lin, I. K.; Wang, H.; Huang, P. P.; Chiang, C. H.; Yang, Y. C.; Wang, I. T.; Du, H. Y.; Wen, C. Y.; Shyue, J. J.; Chen, C. W.; Chen, K. H.; Chiu, P. W.; Chen, L. C., Fast growth of large-grain and continuous MoS_2 films through a self-capping vapor-liquid-solid method. *Nat. Commun.* **2020,** *11*, 3682.

2. Lu, S. C.; Leburton, J. P., Electronic structures of defects and magnetic impurities in MoS_2 monolayers. *Nanoscale Res. Lett.* **2014,** *9*, 2413.

3. Fan, X. L.; An, Y. R.; Guo, W. J., Ferromagnetism in transitional Metal-doped MoS_2 monolayer. *Nanoscale Res. Lett.* **2016,** *11*, 154.

4. Mekonnen, S.; Singh, P., Electronic structure and nearly room-temperature ferromagnetism in V-doped monolayer and bilayer MoS_2. *Int. J. Mod. Phys. B* **2018,** *32*, 1850231.

5. Miao, Y. P.; Li, Y.; Fang, Q. L.; Huang, Y. H.; Sun, Y. J.; Xu, K. W.; Ma, F.; Chu, P. K., Effects of dopant separation on electronic states and magnetism in monolayer MoS_2. *Appl. Surf. Sci.* **2018,** *428*, 226-232.

6. Obodo, K. O.; Ouma, C. N. M.; Obodo, J. T.; Braun, M.; Bessarabov, D., First principles study of single and multi-site transition metal dopant ions in MoS_2 monolayer. *Comput. Condens. Matter* **2019,** *21*, e00419.

7. Gao, Y.; Ganguli, N.; Kelly, P. J., DFT study of itinerant ferromagnetism in *p*-doped monolayers of MoS_2. *Phys. Rev. B* **2019,** *100*, 235440.

8. Zhang, F.; Zheng, B.; Sebastian, A.; Olson, D. H.; Liu, M.; Fujisawa, K.; Pham, Y. T. H.; Jimenez, V. O.; Kalappattil, V.; Miao, L.; Zhang, T.; Pendurthi, R.; Lei, Y.; Elias, A. L.; Wang, Y.; Alem, N.; Hopkins, P. E.; Das, S.; Crespi, V. H.; Phan, M. H.; Terrones, M., Monolayer

vanadium-doped tungsten disulfide: A room-temperature dilute magnetic semiconductor. *Adv. Sci.* **2020,** *7*, 2001174.

9. Deng, J.; Zhou, Z.; Chen, J.; Cheng, Z.; Liu, J.; Wang, Z., Vanadium-doped molybdenum diselenide atomic layers with room-temperature ferromagnetism. *ChemPhysChem* **2022,** *23*, e202200162.

10. Qorbani, M.; Sabbah, A.; Lai, Y.-R.; Kholimatussadiah, S.; Quadir, S.; Huang, C.-Y.; Shown, I.; Huang, Y.-F.; Hayashi, M.; Chen, K.-H.; Chen, L.-C., Atomistic insights into highly active reconstructed edges of monolayer 2H-WSe_2 photocatalyst. *Nat. Commun.* **2022,** *13*, 1256.

11. Lee, C.; Yan, H.; Brus, L. E.; Heinz, T. F.; Hone, J.; Ryu, S., Anomalous lattice vibrations of single- and few-layer MoS_2. *ACS Nano* **2010,** *4*, 2695-700.
